# Evidence for Highly Variable, Region-Specific Patterns of T-Cell Epitope Mutations Accumulating in *Mycobacterium tuberculosis* Strains

**DOI:** 10.3389/fimmu.2019.00195

**Published:** 2019-02-13

**Authors:** Arunachalam Ramaiah, Soumya Nayak, Srabanti Rakshit, Abigail L. Manson, Thomas Abeel, Sivakumar Shanmugam, Pravat Nalini Sahoo, Anto Jesuraj Uday Kumar John, Jagadish Chandrabose Sundaramurthi, Sujatha Narayanan, George D'Souza, Paul von Hoegen, Tom H. M. Ottenhoff, Soumya Swaminathan, Ashlee M. Earl, Annapurna Vyakarnam

**Affiliations:** ^1^Centre for Infectious Disease Research, Indian Institute of Science, Bangalore, India; ^2^Broad Institute of MIT and Harvard, Cambridge, MA, United States; ^3^National Institute for Research in Tuberculosis (ICMR), Chennai, India; ^4^Department of Pulmonary Medicine, St. John's Research Institute, Bangalore, India; ^5^JPT Peptide Technologies GmbH, Berlin, Germany; ^6^Department of Infectious Diseases, Leiden University Medical Center, Leiden, Netherlands; ^7^Department of Infectious Diseases, Faculty of Life Sciences & Medicine, School of Immunology and Microbial Sciences, King's College London, London, United Kingdom

**Keywords:** *Mycobacterium tuberculosis*, genome sequence, T-cell epitopes, evolution, immune response, TB vaccine

## Abstract

Vaccines that confer protection through induction of adaptive T-cell immunity rely on understanding T-cell epitope (TCE) evolution induced by immune escape. This is poorly understood in tuberculosis (TB), an ancient, chronic disease, where CD4 T-cell immunity is of recognized importance. We probed 905 functionally validated, curated human CD4 T cell epitopes in 79 *Mycobacterium tuberculosis* (Mtb) whole genomes from India. This screen resulted in identifying 64 mutated epitopes in these strains initially using a computational pipeline and subsequently verified by single nucleotide polymorphism (SNP) analysis. SNP based phylogeny revealed the 79 Mtb strains to cluster to East African Indian (EAI), Central Asian Strain (CAS), and Beijing (BEI) lineages. Eighty-nine percent of the mutated T-cell epitopes (mTCEs) identified in the 79 Mtb strains from India has not previously been reported. These mTCEs were encoded by genes with high nucleotide diversity scores including seven mTCEs encoded by six antigens in the top 10% of rapidly divergent Mtb genes encoded by these strains. Using a T cell functional assay readout, we demonstrate 62% of mTCEs tested to significantly alter CD4 T-cell IFNγ and/or IL2 secretion with associated changes in predicted HLA-DR binding affinity: the gain of function mutations displayed higher predicted HLA-DR binding affinity and conversely mutations resulting in loss of function displayed lower predicted HLA-DR binding affinity. Most mutated antigens belonged to the cell wall/cell processes, and, intermediary metabolism and respiration families though all known Mtb proteins encoded mutations. Analysis of the mTCEs in an SNP database of 5,310 global Mtb strains identified 82% mTCEs to be significantly more prevalent in Mtb strains isolated from India, including 36 mTCEs identified exclusively in strains from India. These epitopes had a significantly higher predicted binding affinity to HLA-DR alleles that were highly prevalent in India compared to HLA-DR alleles rare in India, highlighting HLA-DR maybe an important driver of these mutations. This first evidence of region-specific TCE mutations potentially employed by Mtb to escape host immunity has important implications for TB vaccine design.

## Introduction

A well-recognized consequence of adaptive immune responses is the occurrence of pathogen immune escape variants arising through positive selection, an effective strategy for pathogens to persist by evading host immunity and creating pathogen diversity (1). In the case of *Mycobacterium tuberculosis* (Mtb), a highly successful bacterial pathogen that has effectively co-evolved with the human host for thousands of years, there is clear evidence for emergence of drug selection variants (2, 3). However, there is little evidence that the host immune response can exert sufficient selection pressure to induce pathogen escape.

Adaptive immune responses, particularly T-cell responses, have been implicated in protective immunity to tuberculosis (TB), with rapid disease progression noted in humans and animal models with T cell-deficiencies (4–6). Among T lymphocytes, there is well-documented evidence that CD4 T cells are essential for protection (7). Thus, HIV-induced CD4 T cell loss is a major predisposing factor for acquisition of active TB, while restoration of CD4 T-cells following antiretroviral therapy reduces this enhanced susceptibility to TB (3). Furthermore, Mtb-specific interferon gamma (IFNγ) and interleukin-2 (IL-2) secreting CD4, rather than CD8 T cells, correlate with protection (8–11). Despite a critical role for specific CD4 T-cells in protective immunity, bacterial persistence in an infected host is common and can lead to subsequent reactivation and transmission of TB. The mechanisms underlying this persistence may include bacterial disruption of antigen presentation and interference with T-cell effector mechanisms (12). In addition, antigenic variation arising from host immune pressure can lead to immune escape; to what extent this contributes to Mtb persistence in an infected host is poorly understood.

Very few studies have probed the evolution of T-cell epitopes (TCEs) encoded in Mtb genomes. The study of Comas et al. (13), initially showed Mtb-specific TCEs to be highly conserved in a small study of 21 strains, despite known continuous diversification of Mtb genomes. This analysis was based on probing the sequence of Mtb strains for conservation vs. (coding) mutations in TCEs curated in the Immune Epitope Database and Analysis Resources (IEDB) (14). More recently the same group confirmed this observation using an expanded collection of 216 Mtb strains (15). Additionally, using a functional genomics approach, they found several new TCEs in proteins that selectively mapped to more rapidly diversifying regions of the Mtb genome that also impacted T-cell interferon gamma release (15), thus providing important functional genetic evidence supporting T-cell immune escape in Mtb infection. The significance of this observation relies on understanding whether TCE evolution is a general phenomenon more commonly found in TB, and, importantly establishing whether similar mutation patterns are reproduced in Mtb strains circulating in other geographic regions of the world. To address this important issue, we first analyzed functionally verified TCEs curated in IEDB in 79 Mtb whole genomes isolated from subjects in India. The mutations identified in these epitopes were then contextualized to corresponding non-synonymous changes using a single nucleotide polymorphism (SNP) database of 5,310 Mtb strains circulating worldwide, that also included the 79 whole genomes from India, as reported in our recent studies of drug resistance mutations in these strains (16, 17). Our current study therefore aims to provide a highly comprehensive analysis to date of TCE mutations in Mtb strains.

We hypothesized that, based on inherent lineage differences combined with population related HLA differences, which can significantly imprint T-cell epitope evolution (18), the pattern, specificity and nature of T-cell epitope mutations in Mtb strains prevalent in India will differ from Mtb strains circulating globally outside India. Evidence is provided in support of this hypothesis.

## Materials and Methods

### Study Population for Isolation of Mtb Strains From India

Extended clinical details including ethics of infected subjects and strain collection is detailed in our previous publications, where 220 strains were studied from India (16, 17). Briefly, patients attending any of the designated microscopy centers in 5 blocks of Tiruvallur district, Tamil Nadu, India between May 1999 and December 2003, with a history of cough of 3 weeks or more were investigated for tuberculosis. Initially, two sputum specimens (one spot and one overnight specimen) were tested by sputum smear microscopy using the Ziehl–Neelsen method (19). Those patients diagnosed to have TB (on sputum smear microscopy or clinical grounds) were categorized (based on previous history of treatment) and initiated on the appropriate treatment regimen by the medical officers at the respective centers [Category I, II, III of the Revised National Tuberculosis Program (RNTCP)]. Their response to treatment was monitored by monthly clinical response as well as sputum smear examination at 2 months and at end of treatment. Two additional sputum samples were collected from these patients for investigations at National Institute for Research in Tuberculosis (NIRT), Chennai, India. Further, patients with suspected drug resistance from all over Tamil Nadu of India were referred to NIRT for drug susceptibility testing. Three sputum specimens were collected at baseline and patients initiated on treatment, based on history and available drug susceptibility testing (DST) (Category IV and V of RNTCP). They were followed every month with a clinical review and sputum examination.

### Bacteriologic Methods and DNA Isolation

The method for bacterial culture and DNA extraction were detailed (16, 17). In brief, sputum samples were transported to NIRT on the same day and were processed for culture on Löwenstein-Jensen medium. Culture positive isolates were selected and subjected to identification, and susceptibility testing. Seventy-nine Mtb isolates were considered for further studies. Resistance to Rifampicin, Isoniazid and Ethambutol was determined by the minimal inhibitory concentration method (MIC), and resistance to streptomycin was determined by the Resistance Ratio (RR) method (20, 21). Genomic DNA was extracted from Mtb cultures by standard CTAB–NaCl extraction method (22). Spoligotyping was performed using standard protocol (23). We analyzed our spoligotyping data on a global scale through comparison with the international SpolDB4 database. All 79 Mtb strains were broadly classified into three groups as follows: drug sensitive (DS): susceptible to all the drugs tested, including streptomycin, isoniazid, rifampicin and ethambutol; Poly-resistant (PR): resistant to more than one first-line anti-TB drug, other than isoniazid + rifampicin; Multi drug resistant (MDR): resistant to rifampicin and isoniazid in addition to any other drug tested (http://www.who.int/tb/areas-of-work/drug-resistant-tb/types/en/).

### Genome Sequencing, Assembly, and Annotation of 79 Mtb Strains

Library preparation and whole genome sequencing (WGS) were performed as previously described (16, 17, 24, 25). All genomes were uniformly annotated by transferring annotations from the reference, *M. tuberculosis* H37Rv. The reference (accession #CP003248.2) was aligned to draft assemblies using NUCmer (26). This alignment was used to map reference genes over to the target genomes. For those genes not clearly mapping to *M. tuberculosis* H37Rv, the protein-coding genes were predicted with the software tool Prodigal (27). tRNAs were identified by tRNAscan-SE (28) and rRNA genes were predicted using RNAmmer (29). Gene product names were assigned based on top Blast hits against the SwissProt protein database (≥70% identity and ≥70% query coverage), and protein family profile search against the TIGRFAM Hmmer Equivalogs. Additional annotation analyses performed include Pfam (30), TIGRFAM (31), Kyoto Encyclopedia of Genes and Genomes (KEGG) (32), clusters of orthologous groups (COG) (33), Gene Ontology (GO) (34), EFICAz (35), SignalP (36), and Transmembrane Helices and Hidden Markov Model (TMHMM) (37).

### Verification to Remove Paralogs

As it is important to ensure that epitopes identified in our study were identified in true orthologs, rather than in closely related paralogous proteins, we used established pipelines to perform orthogroup clustering, including annotation transfer (24) and SynerClust (available at: https://github.com/SynerClust/SynerClust), which is based on the Synergy algorithm (38, 39). This algorithm takes into account both gene similarity and genomic synteny, leading to more accurate placement of paralogs into the correct orthogroups.

Our initial selection of orthologs based on reciprocal blast hits was verified using the Broad's TB annotation pipeline (39) for transferring annotations from Mtb H37Rv. According to this annotation pipeline, genome alignments were performed to map the genes from the H37Rv reference genome onto each newly assembled genome (16, 17). If annotated gene names were available for a particular orthogroup, we verified that the set of genes sharing the same gene name were found in the same orthogroup. For genes without annotated gene names from the annotation pipeline, we used orthogroups calculated using SynerClust.

### Phylogenetic Analysis

Next-generation sequence (NGS) reads from 79 clinical isolates from India were mapped onto a reference strain, H37Rv (GenBank #CP003248.2), using BWA version 0.5.9 (40). In cases in which read coverage of the reference was >200 × , reads were down-sampled using Picard (http://broadinstitute.github.io/picard/) prior to mapping. Positions that varied relative to the reference were identified using Pilon version 1.5 as described in Walker et al. (41). All sites with unambiguous SNPs in at least one strain were combined into a concatenated alignment. Ambiguous positions were treated as missing data. The concatenated alignment was then used to generate a midpoint rooted maximum likelihood (ML) phylogenetic tree in RAxML (version 7.3.3) (42) under a GTRCAT substitution model with 1,000 bootstrap replicates. Epitope information was overlaid on the phylogeny using the Peacock software (43). Each SNP was mapped to a gene locus, and its coding effect was annotated with VCF annotator (44).

### Annotation Based Pipeline for Screening Mutations in T-Cell Antigens and Their Epitopes

IEDB was used as a source to retrieve a total of 1,860 experimentally verified T-cell epitopes (TCEs) of Mtb strains (date 30-Sep-2014). A total of 1,640 out of 1,860 TCEs tested in humans were unique by epitope identification (ID) number, and as a result, the remaining 220 identical cum repeated TCEs were excluded for further analysis. BLASTP searching was done for all 1,640 TCEs against the Mtb reference strain H37Rv to determine identical epitopes and their locus. We identified 1,783 hits with 100% identity. Elimination of epitopes that matched partially resulted in 1,723 hits. Of these, 1,465 were unique by ID number; the remaining 258 hits were duplicates and mapped to more than one antigen (i.e., antigens from repetitive regions include PE/PPE family, transposase, phage protein and integrase) and therefore excluded as independent hits. The identification of the antigens that these 1,465 epitopes covered was verified using the reference strain H37Rv as well as the respective bibliographic reference from IEDB. This resulted in identifying 1,465 TCEs encoded by 363 T cell proteins/antigens of H37Rv. Overlapping epitopes covering a single antigen were next eliminated. This resulted in identifying a total of 1,101 independent TCEs encoded by 363 Mtb antigens from both non-repetitive and repetitive regions with 100% identity to H37Rv (Figure S1) that was used to scan 79 Mtb strains.

In order to extract the orthologous 363 T cell antigens of H37Rv from Mtb strains that we sequenced, stand-alone reciprocal BLAST and in-house Perl and Shell scripts were used. We then used an identical method to scan 1,101 TCEs in these 363 antigens to determine the diversity and evolutionary patterns of TCEs. If a particular epitope mapped with a paralogous antigen, then manual mapping was employed. After excluding the epitopes from repetitive regions, we finally considered only 905 independent TCEs encoded by 298 antigens. We analyzed all 905 human CD4^+^ and CD8^+^ TCEs as one group. However, the majority (82%) were CD4 T cell epitopes (Supplementary File 1A); similarly, 91% of the 64 mutated TCEs (see Results section) were CD4 TCEs (Supplementary File 1B). Notably, all 298 T cell parent/reference antigens, and 40 of 298 mutated antigens (mAg) carrying mutated TCEs (mTCEs), were classified into eight different functional categories using TubercuList (45) (Figure S2).

### Matching mTCE to Global SNP Database and Determining the Global Distribution of mTCE

All mTCE coordinates were converted to the corresponding genomic coordinates, with single amino acid changes corresponding to any one of three positions within the corresponding codon. If an epitope had more than one mutated position, then each position was considered individually; that is, mTCE with two or more mutations were identified by more than one single nucleotide polymorphism (SNP). Mutant is defined as amino acid changes within the 79 Indian isolates identified relative to reference Mtb strain H37Rv or the non-synonymous mutations in the gene where the epitopes are located. A total of 64 out of 69 mTCEs identified matched exactly to 66 SNPs (Supplementary File 2, Sheets 2). We then matched each of the 66 SNPs with the SNPs reported from 5,310 global Mtb strains in our recent study (16, 17) where the methodology for SNP identification is described in detail. In Supplementary File 2, the method used to calculate the global distribution of a given mTCE is provided. In brief, prevalence or relative enrichment was calculated as the percentage of strains from India carrying an mTCE matched SNP (mTCE/SNP) divided by the percentage of strains outside India carrying the same mTCE/SNP. A prevalence >2-fold represented mTCE/SNP more prevalent in India; a prevalence of one, or close to one, represented mTCE/SNP equally distributed between India and outside India, and a prevalence < 0.5 represented mTCE/SNP more prevalent outside India. A proportionality test (Fisher's exact test) adjusted for multiple comparisons identified mTCE that were significantly more prevalent in India vs. outside India using Graph Pad PRISM v7 software. Adjusted *p*-values were calculated using *p.adjust* function of the “R” package with the “BH” correction method (46). If the value of a *p*-value is reported as < 0.0001 its value is taken as 0.0001.

### MHC Class II Binding Predictions

Major Histocompatibility Complex (MHC) (or) Human Leukocyte Antigen (HLA) class II binding predictions were carried out for all mutated 15-mer TCE from 79 Mtb strains and their corresponding parent epitopes from strain H37Rv for known Indian alleles (http://www.allelefrequencies.net/) using widely used IEDB analysis resource consensus tool (47–50) under IEDB recommended prediction methods. The IEDB recommended method works based on availability of predictors and previously observed predictive performance; the IEDB selection tries to use the best possible method for a given MHC molecule. This method uses the Consensus approach, combining NN-align, SMM-align, CombLib, and Sturniolo to determine if any corresponding predictor is available for the molecule; otherwise NetMHCIIpan is used. All 20 15-mer CD4 mTCEs identified and their corresponding parents were tested for predicted binding to 55 well-recognized Indian HLA alleles reported in IMGT/HLA database (51). The HLA-DR alleles employed in this study covered most of the Indian population (52, 53). The binding prediction results are given in units of IC 50 nM, such that a lower number indicates higher affinity. Thus, we considered only those epitopes that were restricted by the given alleles with predicted binding affinity score IC ≤ 10 nM, as these high affinity epitopes are more likely to bind and mount an effective immune response. For the IEDB recommended method, the median percentile rank of the aforementioned methods was used as the representative percentile rank.

### Determining the Binding Affinity of mTCE to HLA-DR Alleles Prevalent in Different Geographical Regions of the World

The HLA binding affinity was predicted for all 15 mer parent TCE (*N* = 20) and their mutants (mTCEs) (*N* = 20) to a total of 50 MHC Class II high prevalent alleles (allele frequency ≥ 0.01) (http://allelefrequencies.net/) that are classified as follows: 16 alleles highly prevalent in India but also prevalent outside India (Group A); 11 alleles less prevalent in India but high prevalent in outside India; and 23 alleles that are not reported or not common in India, but highly prevalent in rest of the world (divided as geographical regions: Australia, Europe, North Africa, North America, North-East Asia, Oceania, South and Central America, South-East Asia, Sub-Saharan Africa and Western Asia; Group B). The latter 23 alleles were reported with high frequency in at least two of ten geographical regions outside India. A total of 20 out of 64 15-mer mTCEs were classified into four groups based on their global geographic distribution in 5,310 Mtb strains as follows: mTCE exclusive to India (*n* = 10); mTCE enriched in India (*n* = 5); mTCE in equal proportion inside and outside India (*n* = 2) and mTCE enriched outside India (*n* = 3) (Supplementary File 4).

### Clinical Samples to Verify Biological Activity of mTCEs

A total of 130 individuals were prospectively recruited between April 2015 and April 2016 at St. John's Medical College and Hospital and 30 ml of peripheral blood was collected from them by venipuncture in anticoagulant (ACD/Heparin/EDTA) depending on the immune assays as part of a Center of Excellence Grant on HIV-TB Coinfection awarded to AV. Out of 130 subjects recruited, 42 were pulmonary TB patients and the rest 88 healthy individuals were screened by the IGRA test. Of these, 26 were IGRA^+^ and the remaining IGRA^−^ served as uninfected healthy controls. For this study, blood and peripheral blood mononuclear cell (PBMC) from 13 IGRA^−^, 12 IGRA^+^, and 11 PTB subjects was available for T cell functional assays. Clinical information of study participants is summarized in a Table S2 and a detailed clinical file of samples used for this study is provided in Supplementary File 5. Three clinical groups were tested for responses to TCE identified in this study: (i) IGRA^−^, (ii) IGRA^+^ or latent TB infection (LTBI), and (iii) pulmonary TB (PTB).

#### IGRA^−^ and IGRA^+^ Subjects

Health Care workers of St. John's National Academy of Health Sciences were recruited through internal calls highlighting the nature and importance of the study. Study subjects were classified as IGRA^+^ based on a standard QuantiFERON TB Gold In-tube test (Qiagen, Germany) results (54). IGRA^+^ subjects were classified as LTBI if they had not received preventive/curative therapy for TB in the past. A total of 88 subjects were recruited and screened by the IGRA test. Of these, 26 were IGRA^+^ and the remaining IGRA^−^ served as uninfected healthy controls, of which 12 IGRA^+^ and 13 IGRA^−^ were used in our study for different assays. Thirteen IGRA^−^ subjects included for the study of which 46% were males with a median age of 27 years (range 20–39 years). Twelve IGRA^+^ subjects were included for the study of which 75% were males with a median age of 32 years (range 25–73 years).

#### Pulmonary TB

Subjects for this prospective study were enrolled from the Revised National Tuberculosis Program (RNTCP) clinic of St. John's Medical College and Hospital. A diagnosis of pulmonary TB was ascertained by sputum smear microscopy and culture. Standard smear grading of 1^+^, 2^+^, and 3^+^ was used to ascertain the bacterial burden. Smear negative cases of pulmonary TB cases were diagnosed and classified by the treating clinician from plain chest radiographs as per the RNTCP standards. All subjects were treatment naive at the time of enrollment. Consenting adult patients meeting the above inclusion and exclusion criteria were included. Sputum samples were collected for reconfirmation of TB diagnosis by GeneXpert MTB/RIF assay (Cephid, USA). A total of 11 cases of pulmonary TB were included in the study of which 90.9% were males with a median age of 37 years (range 22–66 years).

### IFNγ Whole Blood Assay

Peptides were synthesized by JPT Peptide Technologies Ltd (Berlin, Germany) and reconstituted in dimethyl sulfoxide (DMSO). Stocks were diluted to 6.25 mg/ml and stored at −20°C in aliquots. Within 3–4 h of blood draw, heparinized whole blood obtained from healthy IGRA^+^ individuals and active TB patients was diluted to a final concentration of 1:5 with RPMI 1,640 medium and 100 μl of blood/well was transferred to 96 well-round-bottom microtiter plates that were previously loaded with 100 μl each of the candidate peptides at a final concentration of 10 μg/ml. TB-specific antigens, purified protein derivative (PPD; Staten Serum Institute, Denmark), and ESAT6/CFP10 fusion protein (55) were added at a final concentration of 10 μg/ml. Phytohemagglutinin (PHA; 5 μg/ml) and culture medium (RPMI 1,640) were used as positive control and negative control, respectively. Plates were incubated in a 5% CO_2_ incubator at 37°C for 7 days, and culture supernatants were harvested and stored at −80°C for further analysis (56). IFNγ levels were measured in culture supernatants using the OptEIA™Human IFNγ ELISA set (BD Biosciences, UK). Assay background and determining non-specific effects of peptides: Data in Figure S4B shows background unstimulated values to be <2pg/ml (median 0, range: 0–2.3 pg/ml) and were subtracted from all stimulated values. IGRA^−^ subjects were included to highlight the non-specific response to PPD but specific response to ESAT6/CFP10. Peptides for this assay were selected based on either the parent or mutant peptide, when individually tested, inducing a detectable response over background in all IGRA^+^ subjects tested. Cells were stimulated with this peptide pool at 1 μg/ml final concentration for 7 days and background unstimulated control values were subtracted.

### Epitope Specific Responses by Intracellular Cytokine Staining (ICS) Assay

ICS assay with antigen-stimulated PBMCs was performed as previously described (57). Cryopreserved PBMCs were taken out in ice and rapidly thawed in 37°C water bath, transferred to 15 ml tube containing ~3 ml PBS and centrifuged at 2,000 rpm for 5 min at RT. 1 × 10^6^ cells were resuspended in 200 μL medium [RPMI-1640 (GIBCO, Invitrogen) with 10% FCS (GIBCO), 100 U/ml penicillin, 100 μg/ml streptomycin, (SIGMA)], seeded in 96-well round-bottom plates (Costar) and rested for 2 h. Next PBMCs were either left unstimulated (negative control) or stimulated with parent and mutant peptides (10 μg/ml), or positive control PHA (1 μg/ml), in the presence of co-stimulatory antibodies (anti-CD28/CD49d, at 0.5 μg/mL; BD Biosciences) at 37°C and 5% CO_2_. After 4 h of incubation, brefeldin A (10 μg/ml) was added. Next day, PBMC were washed after incubation with EDTA and stained with 5 μl Live/Dead Aqua (Invitrogen) and incubated for 10 min at 4°C in the dark. Cells were washed and fixed for 20 min with 100 μl 1X FACS lysis buffer and permeabilized with 200 μl 1X BD Perm/Wash buffer for 20 min. PBMCs were washed and stained with 50 μl of intracellular staining cocktail containing antibodies (BD Biosciences) (CD3 BV570, CD4 BUV397, CD8 BV711, IFNγ-V450, IL2-APC, TNFα-FITC, IL17A-BV605, and MIP1β-PE) for 30 min at RT in the dark. Cells were washed with 150 μl Perm/Wash buffer and resuspended in ~100 μl 1% paraformaldehyde (Electron Microscopy Sciences). Stained samples were acquired with a standard stopping gate set at 100,000 CD3 lymphocytes on a BD FACSAria^TM^ Fusion flow cytometer using the BD FACSDiva^TM^ version 8.0.1 software. Data was analyzed using FlowJo version 9.9.4 software (TreeStar). Assay background and determining non-specific effects of peptides is shown in Figure S5A. Background unstimulated median CD4 T cell IFNγ and IL2 frequencies were 0.05 and 0.07%, respectively, range 0.03–0.08%. Six IGRA^−^ subjects tested showed a very weak response to a set of five peptides tested (parent median 0.02%, range 0–0.175%; mutant median 0.0365%, range 0–0.165%) (Figure S5B), that induced a strong response in IGRA^+^ subjects tested in parallel confirming that these peptides had negligible non-specific cytokine inducing effects in this assay.

### Data Availability

The sequences have been submitted at the NCBI Sequence Read Archive under BioProject ID PRJNA235852.

## Results

### Novel Patterns of T Cell Epitope Mutations in Multiple Mtb Strains From India

The availability of an extensive collection of functionally validated Mtb-specific T-cell epitopes, including those that map to highly immunodominant (IMD) antigens (58), allowed us to assess, for the first time, mutations within these epitopes in a total of 79 Mtb strains in India. Based on inherent genetic differences in circulating Mtb lineages in South-East Asia and other regions of the world (16, 17), we identified the number and nature of these mutations to differ from those found in a recent study (15). We identified mutated TCEs by conventional approaches as previously reported (13, 15) that used a combination of a whole genome assembly-based methodology and an SNP-based approach. Mutations were defined as amino acid changes within the 79 Indian isolates identified relative to reference Mtb strain H37Rv that comprised non-synonymous mutations in the gene spanning a given epitope. We identified a total of 138 mTCEs by screening 79 Mtb whole genomes from India that were part of a larger study that characterized some 5,310 global Mtb strains (16, 17). Excluding 69 mTCE in the highly repetitive, difficult to sequence PE and PPE gene families as previously reported (13, 15), reduced this number to 69 mTCEs from 43 antigens. Sixty-four of these Sixty-nine mTCEs (93%) were also identified using an SNP-based approach (16) (Figure S1; Supplementary File 2), and verified to be encoded by the correct orthologous genes (see methods). The nature of mutations in these 64 high confidence mutated TCEs encoded by 40 antigens (**Figure 3A**) in the 79 strains circulating in India was comprehensively analyzed as described below.

**Figure 1:**
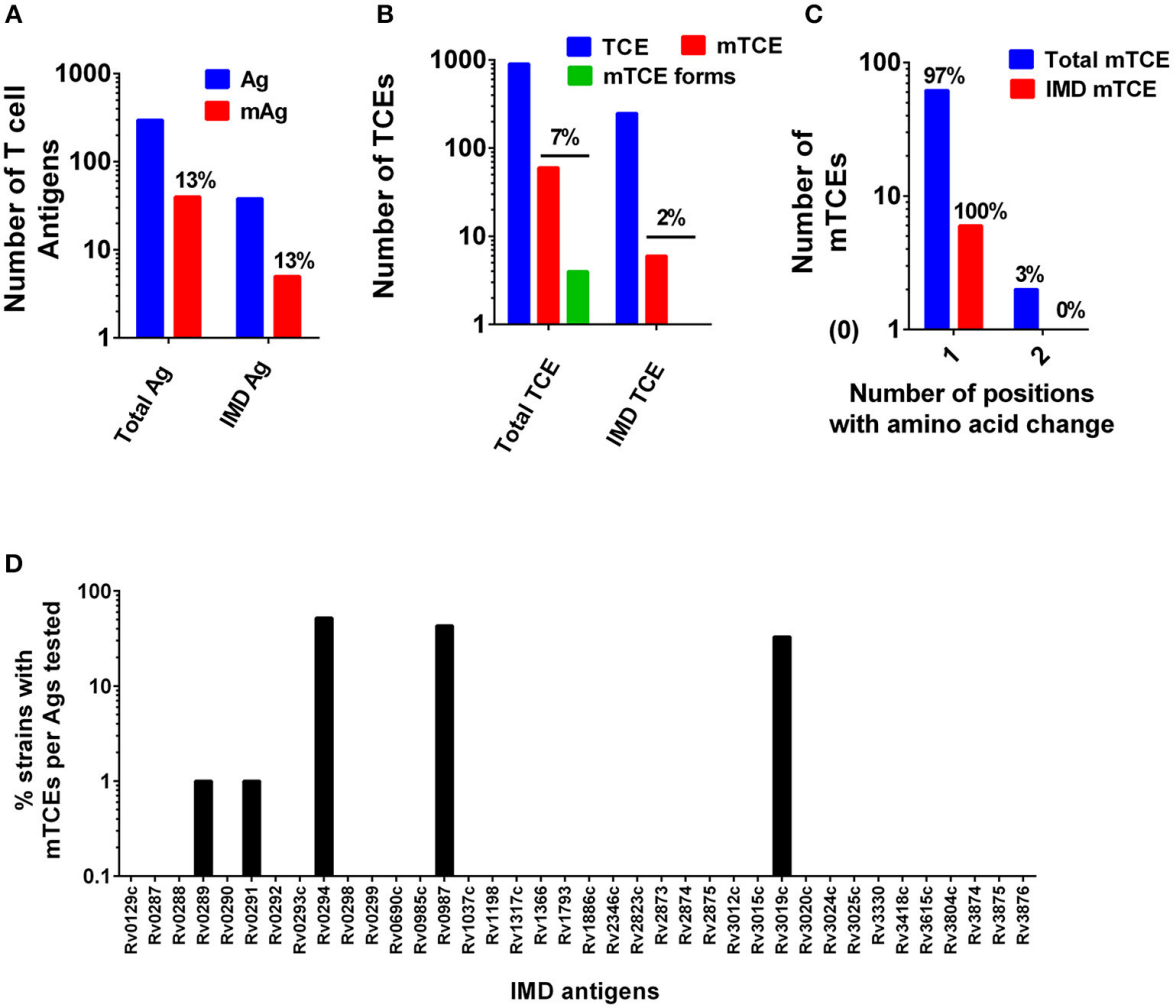
Patterns of mutated antigens (mAg) and mutated epitopes (mTCEs) across 79 Mtb strains. The set of T-cell antigens and epitopes, as well as a subset of immunodominant (IMD) antigens and epitopes, were analyzed based on their nature and locus in the Mtb reference strain H37Rv genome. **(A)** The total number of reference/parent T cell antigens (blue), the number of mAg (red), and the number of parent immunodominant antigens and mutants analyzed is shown. **(B)** The total number of reference/parent T-cell epitopes (blue), the number of mTCE (red), and the number of immunodominant parent T-cell epitopes and mutants are shown. The green bar identifies mTCE with two or more mutant forms. **(C)** The number of mTCE with one vs. two amino acid changes is shown (blue bar represents total mTCE; red bar immunodominant mTCE). **(D)** Representation of mTCEs in immunodominant antigens: A total of 33 of 82 antigens identified by Lindestam et al. (58) was confirmed to be represented in the list of 298 reference antigens analyzed. Bars identify frequency of five mutated immunodominant antigens in 79 Mtb strains.

The overall level of mutations at the antigen and corresponding epitope level was determined (Figures 1A B, respectively, **Figure 3A**). In addition, the proportion of mutations in a subset of 33 recently identified immunodominant antigens (58) was also assessed (Figure 1C). At the antigen level, 15% (5/33) overall mutation frequency was noted (Figure 1A), whereas the proportion within immunodominant TCEs was low (2%, 6 out of 248 TCE's encoded by the 33 immunodominant antigens). Although only a low proportion of epitopes encoded by these immunodominant antigens was mutated, the overall proportion of mTCE relative to all 905 parent epitopes analyzed was higher (7%, 64/905), reflecting focused mutations within few immunodominant antigens (Figure 1B). Thus, only five of the 33 immunodominant antigens carried mutations encoding 6 mTCEs (Figure 1D). Among five, three of these immunodominant antigens belonged to the “cell wall and cell processes” functional category (Rv0289/espG3, Rv0987, Rv3019c/esxR), and two belonged to the “intermediary metabolism and respiration” category (Rv0291/mycP3, Rv0294/tam).

A number of features of the mutated TCE identified are novel: (1) Although 97% of mutations represented single amino acid changes, 3% of mTCEs contained two amino acid changes (Figure 1C) (2) Two mTCEs had at least two different mutant forms (*n* = 4) (Figure 1B); these mTCEs are of specific interest as multiple variants of a single epitope can arise due to immune selection pressure with functional consequences (see **Figure 4**). (3) The 64 mTCEs identified spanned all known Mtb functions (45), of which 44% belonged to the “cell wall and cell processes” functional category (Figure S2), in keeping with the proportion of parent epitopes in each category. (4) The availability of detailed clinical records enabled us to interrogate if mTCE patterns were skewed by the clinical stage of the infected host. Figures S3A–E reveals remarkable consistency in the number of mTCE that arise within the five clinical groups analyzed, with a median of 7 mTCEs per strain tested, each of which was isolated from a single infected donor [range 5–13]. Importantly, none of the 79 strains analyzed lacked an mTCE, of which 19% were common to all clinical groups (Venn Diagram, Figure S3F). (5) In addition, we were able to interrogate the stability of intra-donor mutations and demonstrate in an analysis of six subjects, that multiple strains [range 2–5 strains] isolated from a single donor over several time points consistently resulted in identifying exactly the same TCE mutations (Supplementary File 6).

**Figure 2:**
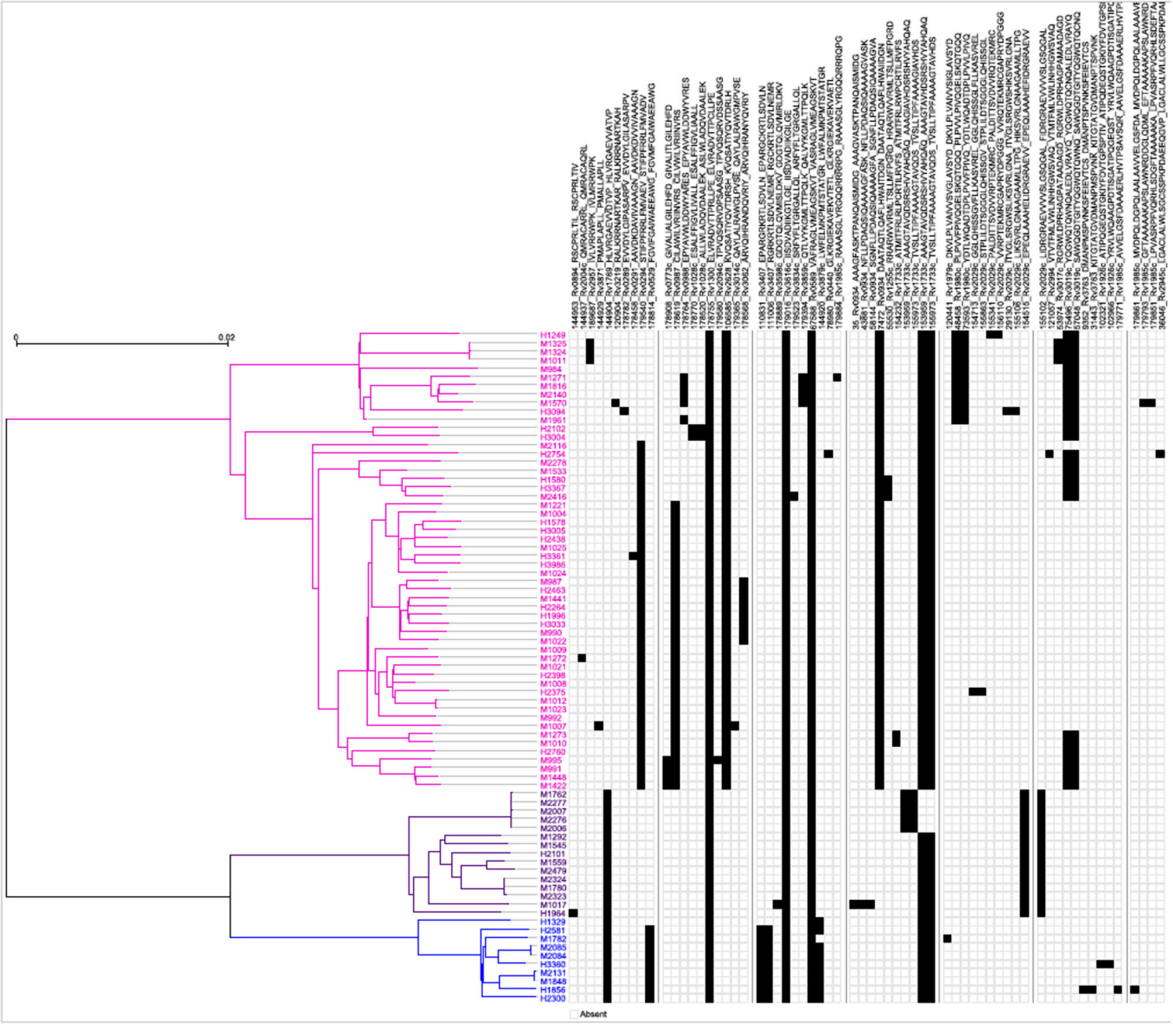
Schematic representation of the phylogenetic relationship of mTCEs of all 79 Mtb strains. Maximum Likelihood tree shows the phylogenetic relationship of the 79 strains studied, based on SNP data. The phylogeny shows two major clades, one consisting of 54 EAI lineage strains (pink), and another one consisting of two clusters, including 10 Beijing/BEI (blue) and 15 CAS (purple) lineage strains, following the naming convention in Comas et al. (13). Each of the columns represents one of the 64 mutated T-cell epitopes. The presence of a mutation in a particular strain is represented by a black square.

Next, a genome-wide SNP-based phylogenetic map of the 79 Mtb strains (y-axis) was constructed and correlated with the 64 mTCEs identified in the 79 strains from India (see x-axis list for mTCE ID) (Figure 2). These 79 Mtb strains from India grouped into three lineages: East African Indian (EAI) (*N* = 54), Central Asian Strain (CAS) (*N* = 15), and Beijing (*N* = 10), with each black square on the map representing an mTCE. Fifty percent (32/64) of the mTCEs occur in a single strain (represented by a single black square per column). Of the 64 mTCEs, 91% (*N* = 58) were lineage specific. Only five of the 64 mTCEs identified (Epitope ids: 67588, 153959, 155973, 178755, 179016), were common to all three lineages, present in 90–100% strains and therefore highly prevalent (Table S1). Importantly, only 3 of the 64 mTCE identified were previously reported in the large Coscolla et al. (15) study that analyzed 216 Mtb strains (15). Taken together, these data confirm the specificity of TCE mutations in Mtb strains circulating in India to differ markedly from those recently reported in the rest of the world (16). Given that 91% of mTCE we report are lineage specific (Figure 2), inherent phylogenetic differences between the strains we analyzed and those previously studied (15), may be one major factor contributing to this difference.

### Evidence for T-Cell epitope Mutations in Rapidly Diversifying Proteins of Mtb Genomes From India

**Figure 3:**
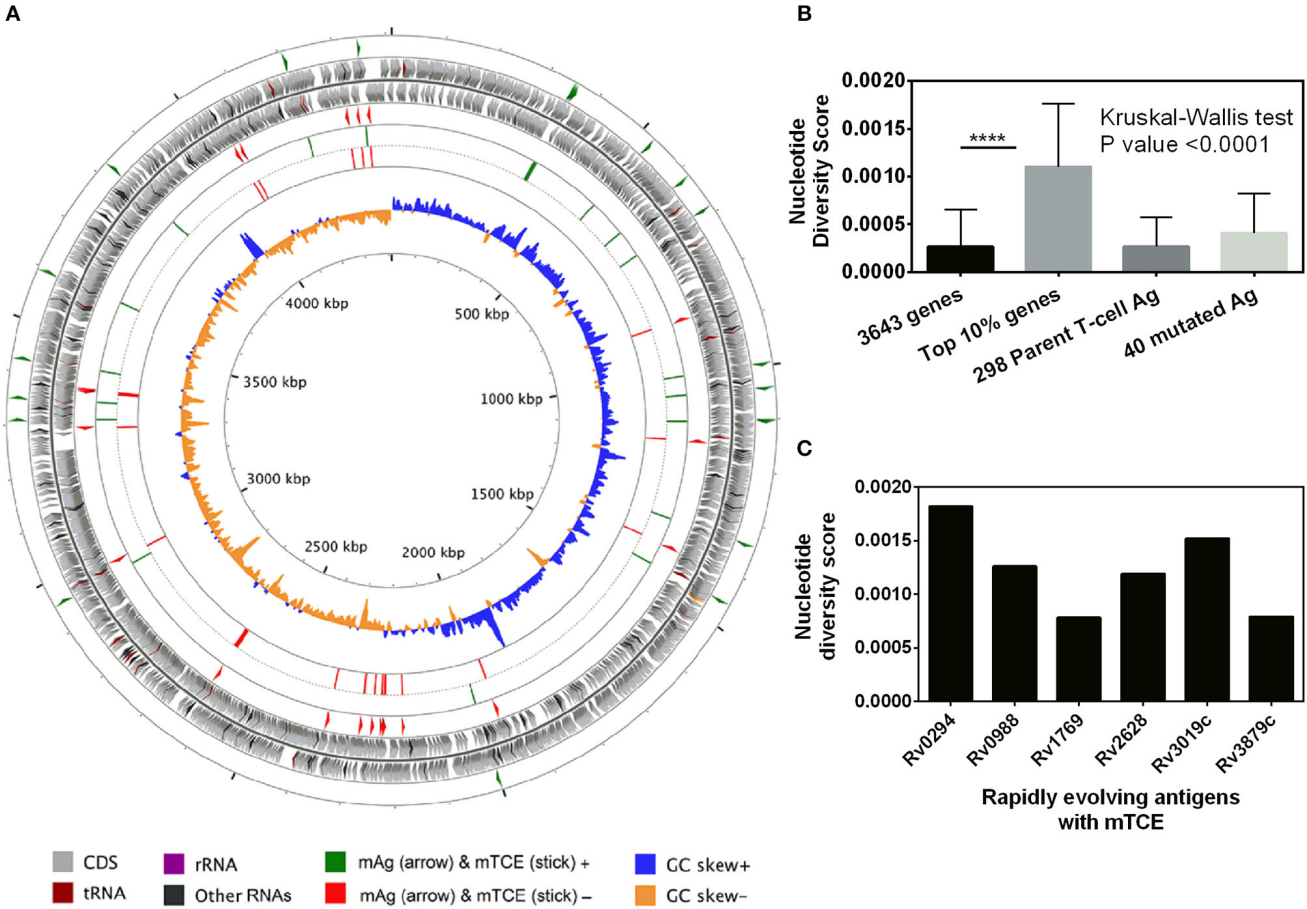
Genetic diversity of 79 Mtb strains from India. **(A)** The scale of the chromosome (in nucleotides, according to the reference Mtb strain H37Rv) is labeled on the innermost ring. From the outer to the inner concentric circle of the chromosome: Circles 1–2 and 3–4, genes with forward and reverse directions are indicated as right and left arrows, respectively. Circles 1 (green) and 4 (red) exemplify the locus of 40 mutated T-cell antigens, which encode mutated T-cell epitopes identified in this study. Circles 2–3 (gray) depict all genes from H37Rv. Circles 5 (green) and 6 (red) show the locus of 64 mutated T-cell, epitopes in the forward and reverse strands, lining their respective genes in circles 1 and 4, respectively. Circles 7 and 8 represents the GC skew of all the genes of H37Rv. **(B)** Bar graph showing nucleotide diversity scores of genes from different categories (^****^p-value < 0.0001). **(C)** Bar graph showing nucleotide diversity scores of six genes encoding mTCEs that fall within the top 10% of nucleotide diversity scores across all 3,643 genes (excluding repetitive genes) encoded by the 79 Mtb strains from India, and therefore designated as rapidly evolving.

Beyond phylogeny, host driven immune selection pressure can influence T-cell epitope changes. Typically, such changes would be expected to occur in rapidly evolving regions of a pathogen genome. To probe this possibility, we determined the nucleotide diversity scores of all 4,038 proteins encoded by the 79 Mtb genomes from India (59), that have already been verified and published (16, 17) as part of a larger study that characterized 5,310 global Mtb strains. In addition, we rigorously excluded genes from repetitive regions in our screen of mutations; so as to consider only those mutations with highest confidence, as reported previously (13, 15). The median nucleotide diversity score of the top 10% of remaining genes in the 79 Mtb whole genomes from India (*N* = 364; excluding repetitive genes) was 0.00095 (range 0.00068–0.01006) (Figure 3B; Supplementary File 3). Interestingly, this was higher (*p* < 0.0001, Mann-Whitney test) than the nucleotide diversity score (median 0.00081; range 0.00060–0.00459) of the top 10% of diverging genes in 216 Mtb strains previously studied (15). The nucleotide diversity score of the 40 antigens encoding all 64 mTCEs identified was marginally higher than the total 298 parent T-cell antigens considered (Figure 3B). We determined if the top 10% of diversifying genes in the 79 strains studied encoded any of the 64 mTCEs identified. Within this set, 6 antigens were identified with an exceptionally high median nucleotide diversity score (Figure 3C), encoding 7 mTCEs. These seven mTCEs were therefore identified to be the most rapidly evolving of the 64 mTCEs identified (Table 1; Supplementary File 3).

**Table 1:** List of known T-cell antigens carrying mutated epitopes present in the top 10% of rapidly evolving genes of 79 Mtb strains isolated in India.

**Rv Number**	**Gene name**	**Protein name**	**Functional category**	**# mTCE**	**Proportion of strains carrying mTCE**	**Lineage**	**Nucleotide diversity score**
Rv0294	Tam	Trans-aconitate methyltransferase	Intermediary metabolism and respiration	1	52	EAI	0.00182
Rv0988	Rv0988	Hypothetical protein Rv0988	Cell wall and cell processes	1	6	EAI	0.00126
Rv1769	Rv1769	Hypothetical protein Rv1769	Conserved hypotheticals	1	32	CAS, BEI	0.00078
Rv2628	Rv2628	Hypothetical protein Rv2628	Conserved hypotheticals	1	68	EAI	0.00119
Rv3019c	esxR	ESAT-6 like protein EsxR	Cell wall and cell processes	2	33	EAI	0.00152
Rv3879c	espK	ESX-1 secretion-associated protein EspK	Cell wall and cell processes	1	11	BEI	0.00079

### Evidence for Functional Impact of Identified mTCEs

Immune selection driven T-cell epitope mutations invariably impact the functional response. We successfully synthesized and screened 36 out of the 64 mutated TCE identified (56%) in a functional assay. The immunogenicity of the novel T cell mutant peptides and their respective naturally occurring parental sequences, which spanned all functional categories of Mtb proteins, were evaluated by quantifying IFNγ in cell culture supernatants after antigenic stimulation of whole blood samples from IGRA^+^ or LTBI and active PTB patients. We first screened all 36 peptides for their capacity to induce IFNγ release measured by ELISA, with a cut-off for a positive IFNγ response set at 5 pg/ml based on assay detection limits (Figure S4A). 32/36 (89%) parent-mutant peptide pairs tested induced a IFNγ response in IGRA^+^ subjects with latent TB compared to only 12 of these pairs (33%) inducing a response in subjects with PTB (Figure S4A). 17/36 (47%) peptide pairs induced a response in > = 25% of the IGRA^+^ donors tested. 11/17 (65%) of these mTCE had a differential response compared to the parent peptide (Figure S6).

In contrast to the response induced in IGRA^+^ subjects, only 7 of 36 peptide pairs (19%) had a response frequency of 25% or higher in PTB subjects. This lack of response in PTB subjects likely reflects specific impairment of epitope-specific responses as the same PTB subjects responded efficiently to an immunodominant recombinant TB antigen, ESAT6/CFP10, similar to a PHA positive control (Figure S4B). This is consistent with data showing subjects with PTB to have compromised blood T cell responses to several Mtb antigens, including latency antigens whilst preserving responses to few immunodominant secreted Mtb antigens, especially at the site of infection in the lung (8, 9). Low frequencies of circulating blood Mtb-specific T cells could therefore be one reason for the lack of response to a broad range of parent and mutant T cell peptides in PTB subjects reported in our study.

**Figure 4:**
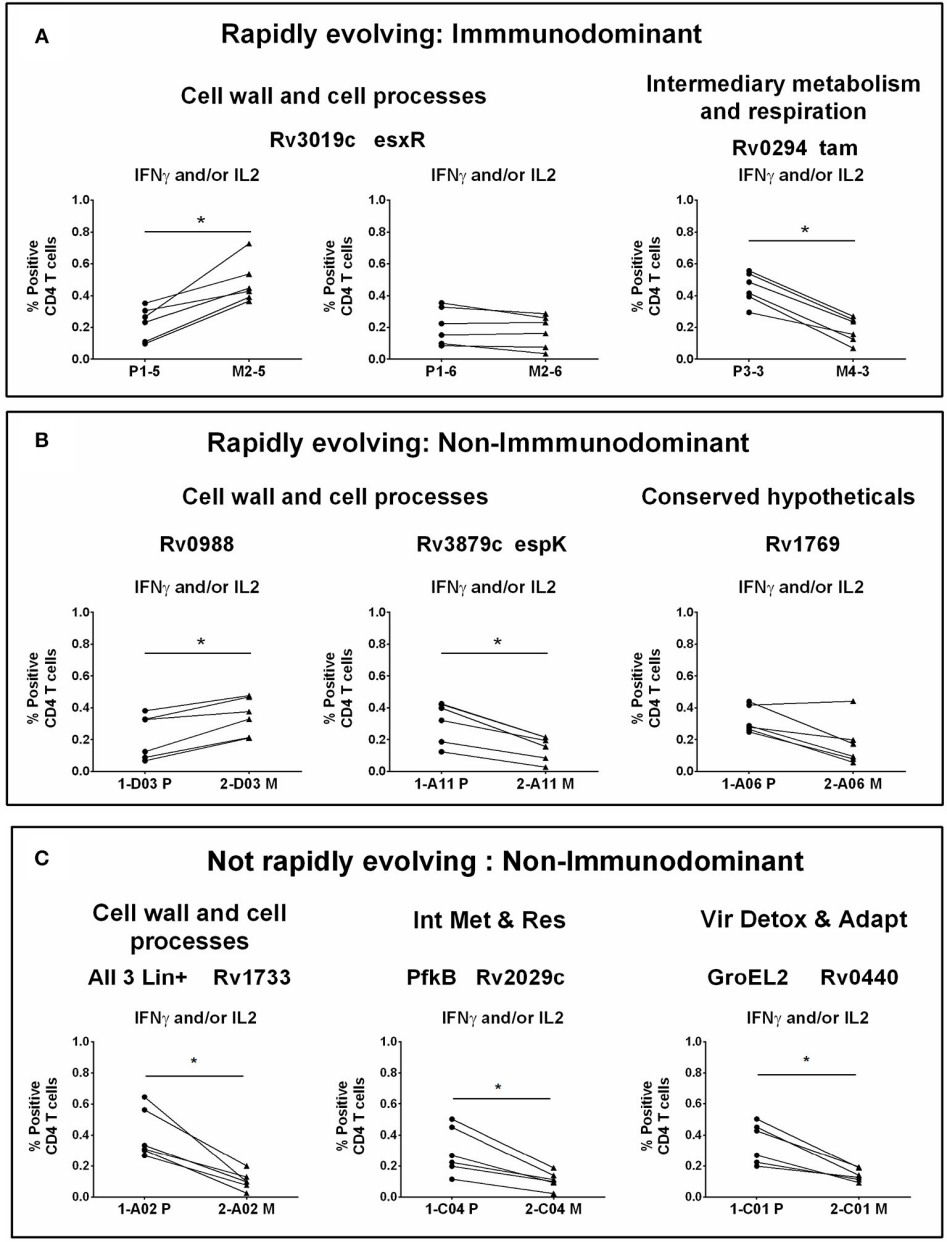
CD4 T cell responses to parent and mutant peptides. PBMCs from IGRA^+^ subjects were stimulated overnight with parent and mutant peptides that are either rapidly evolving or not-rapidly evolving and belonging to immunodominant and non-immunodominant category. Frequencies of IFNγ^+^ and/or IL2^+^ CD4 T cells were determined by ICS assay (see methods). Line graphs shows IFNγ and/or IL2 CD4 T cell frequency following stimulation with a set of nine parent and mutant peptides as individual plots in IGRA^+^ subjects after subtraction of background unstimulated control. Each line is a donor. Statistical analysis was performed using nonparametric Wilcoxon matched-pairs signed rank test. ^*^P < 0.05 was considered significant. mTCE were grouped as follows: **(A)** Rapidly evolving and immunodominant Rv3019c (EsxR) and Rv0294 (tam). **(B)** Rapidly evolving and non-immunodominant Rv0988, Rv3879c (espK) and Rv1769. **(C)** Not rapidly evolving and non-immunodominant Rv1733, Rv2029c, and Rv0440.

We next screened 9 of the peptide pairs that induced a marked IFNγ response in IGRA^+^ subjects (marked with a pink arrow—Figure S4A) by the more sensitive ICS to confirm that the peptides indeed induced CD4 T cell responses using PBMCs from IGRA^+^ subjects. At first, frequencies of IFNγ and/or IL2 expressing CD4 T cells were measured in response to stimulation with these 9 peptide pairs that represented diverse functional categories. Relative to corresponding parent peptides, 2 mTCE induced a significantly higher response; responses to 5 mTCE was significantly impaired and responses to 2 mTCE did not significantly differ from parent (Figure 4). These same set of peptides either totally failed to induce responses or induced very weak responses in IGRA^−^ subjects, highlighting specific recognition of these epitopes by infected subjects (see representative FACS plot and a graph depicting summarized response to set of five peptides, Figure S5).

Out of the 9 peptides tested, 6 were rapidly evolving with 3 each from the immunodominant and non-immunodominant categories. Importantly, EsxR (Rv3019c), an immunodominant antigen belonging to the functional category cell wall and cell processes and is rapidly evolving, encoded two mTCE with one mutant form each; out of the two only one (M2-5) but not M2-6 induced significantly higher responses than their respective parent peptide, (P1-5 and P1-6) (Figure 4A). In contrast, another mTCE of the intermediary metabolism and respiration functional category and immunodominant antigen Tam (Rv0294, M4-3), which is also rapidly evolving elicited lower frequencies of IFNγ and/or IL2 compared to the parent peptide (P3-3, Figure 4A). Next we tested three rapidly evolving non-immunodominant peptides, of which two mTCEs belonging to functional category cell wall and cell processes, had opposing effects on the CD4 T cell response, whereby Rv0988 (2-D03 M) and Rv3879c (espK, 2-A11 M) displayed significantly enhanced and decreased frequencies, respectively. On the other hand, mTCE encoded by Rv1769 (2-A06 M), a conserved hypothetical protein did not exhibit any loss or gain in the overall CD4 T cell response as reflected by the insignificant change in frequencies of IFNγ and/or IL2 CD4 T cells compared to the parent peptide (Figure 4B). Additionally, three more non-immunodominant peptides that are not rapidly evolving were also tested by the ICS assay. Rv1733 (2-A02 M) common to all three lineages and Rv2029c (pfkB, 2-C04 M), two well-known DosR regulon encoded latency antigens (60) (Figure 4C) significantly reduced IFNγ and/or IL2 frequencies relative to parent. Previous studies show latency antigens (Rv1733, Rv2029, and Rv2628—we report on novel mTCE in all three of these antigens) to be better recognized by subjects with latent TB compared to those with disease (8, 61–64). Similarly, the other mTCE of GroEL2 (Rv0440, 2-C01 M) protein belonging to the functional category virulence detoxification and adaptation, also significantly impaired responses compared to the parent CD4 T cell responses by 9 of these peptides tested above concurred with the whole blood IFNγ release assay (data shown only for 2-D03 M and M4-3 in Figure S6). In order to further investigate if any of these peptides induced an altered cytokine response, we tested all 6 rapidly evolving peptides for regulation of a further three cytokines: TNFα, IL17A, and MIP1β. We demonstrate that changes in frequencies of the three additional cytokine-positive CD4 T cells (Figure S7) followed exactly the same pattern as IFNγ and/or IL2 shown in Figures 4A,B for these 6 peptides. Taken together, these data confirm that 7/9 mTCE tested in the ICS assay have a significant functional consequence on Mtb-specific CD4 T cell responses.

### Changes in the Predicted Binding Affinity of mTCE to Common Indian HLA-Class II Alleles Is Associated With mTCE-Induced Changes in T-Cell Responses

**Figure 5:**
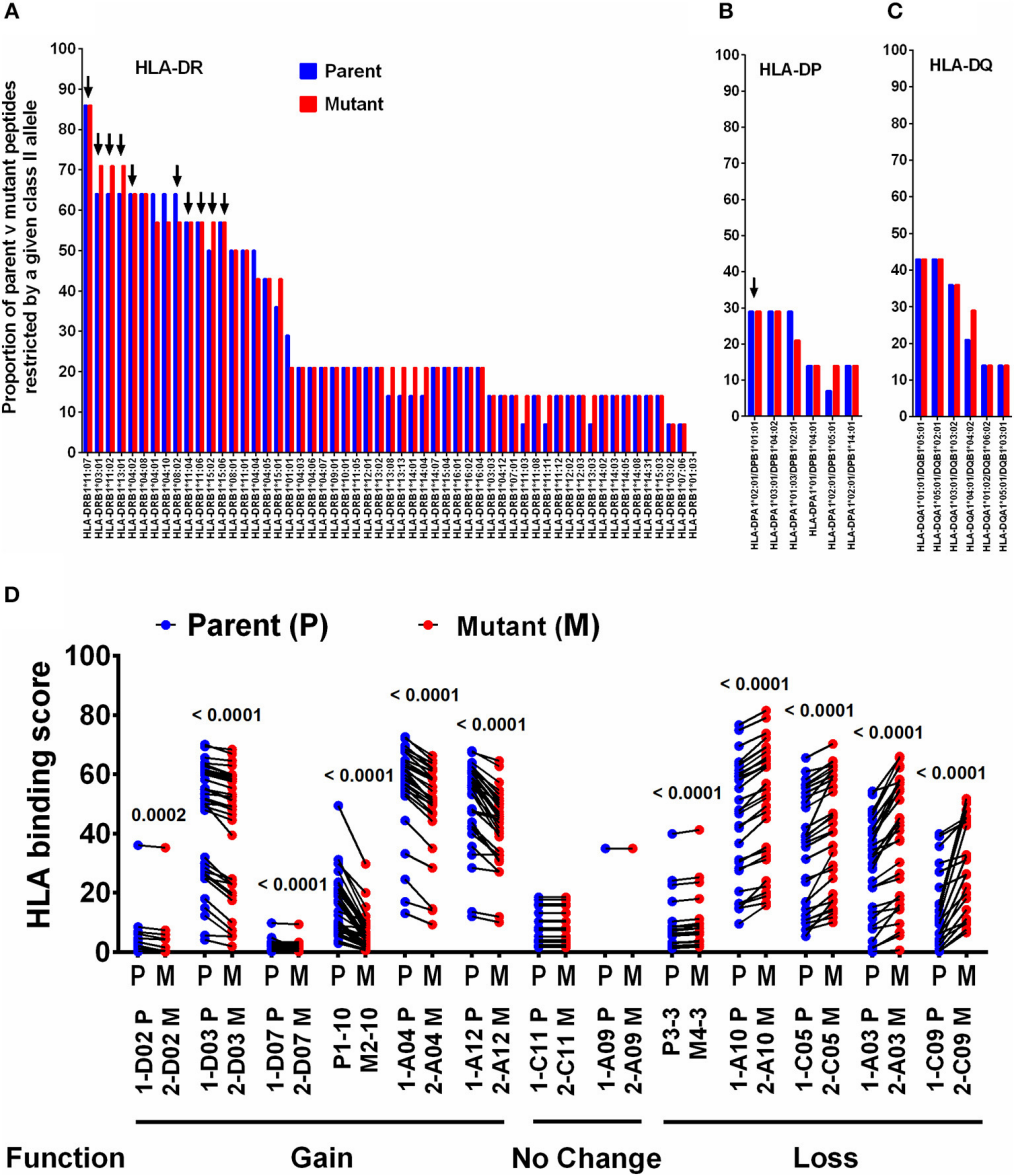
Binding of T-cell epitopes to Indian HLA-DR/DP/DQ alleles. **(A)** Proportion of a 20 mutant TCEs (15 mer; red) and their corresponding parent/reference TCEs (blue) restricted by 55 HLA-DR alleles identified in the Indian population. Only epitopes with binding affinity score IC≤10 nM were considered. **(B)** Binding to DP alleles and, **(C)** Binding to DQ alleles. **(D)** Binding affinity of 13 selected 15 mer parent and mutant peptides to DR alleles, which predict gain or loss or no change in function–see Figure S6 for corresponding IFN**γ** release data.

HLA is a major driving force in shaping immune escape, and therefore T-cell mutations. We therefore predicted that changes in HLA Class-II binding affinity of mTCEs may correlate with functional changes in the immune assays described in Figure 4. Therefore, an *in silico* analysis of the binding affinity of the identified mTCEs to 55 common Indian HLA-DR and 6 DP and 6 DQ alleles, was conducted, using the well-known IEDB analysis resource consensus tool (47–50) which is programmed to analyse peptides/TCEs that are 15-mer in length. A total of 20/64 mTCE were 15-mers. These were analyzed using a binding score of 10 as a cut-off (IC ≤ 10 nM), to identify those with high binding affinities for HLA Class-II alleles and therefore more likely to have a functional consequence. Consistent with publications (65–67), we show that all 20, 15-mer Mtb mTCE/parent peptides tested were predicted to bind to multiple HLA-DR alleles. In this *in silico* analysis, 10 HLA-DR alleles bound >55% peptides with high affinity (see Figure 5A: arrow identifies alleles that are predicted to bind rapidly evolving mTCE). In contrast only a few peptides bound DP (*N* = 5/20) and DQ (*N* = 8/20) alleles with high affinity (Figures 5B,C). Most mTCEs showed improved HLA–DR binding relative to parent (red bar Figures 5A–C).

We next determined if changes in HLA-DR binding of mTCE (relative to parent) were associated with functional changes. Out of 36 peptides synthesized and tested for function, only 13 were 15-mer peptides (Supplementary File 2; Sheet 2). In Figure 5D, we demonstrate the changes in HLA-DR binding of these 13 mutant peptides relative to its parent. The data highlights changes in the binding affinity pattern of each mTCE to be strikingly similar to the functional data shown in Figure S6 for these same 13 peptides. Thus, mTCE: 2-D02 M, 2-D03 M, 2-D07 M, M2-10, 2-A04 M, and 2-A12 M induced higher T-cell responses (Figure S6A) concomitant with increased Class II binding affinity reflected by a significantly reduced binding score compared to parent (Figure 5D). In contrast, mTCEs M4-3, 2-A03 M, 2-A10 M, 2-C05 M, and 2-C09 M displayed HLA binding scores that were significantly enhanced compared to parent, consistent with reduced T cell responses (Figure S6B), associated with decreased Class II binding affinity (Figure 5D). Moreover, mTCE 2-A09 and 2-C11 M, which did not induce significantly different T-cell responses to parent (Figure S6C) bound fewer Class-II alleles with minor differences in binding affinity between parent and mutant (Figure 5D). Taken together these data highlight that mutations in the T-cell epitopes identified can potentially impact binding to HLA-DRB1 alleles in particular.

### Evidence for HLA Class II Selection of Region-Specific mTCE in Mtb Strains From India

**Figure 6:**
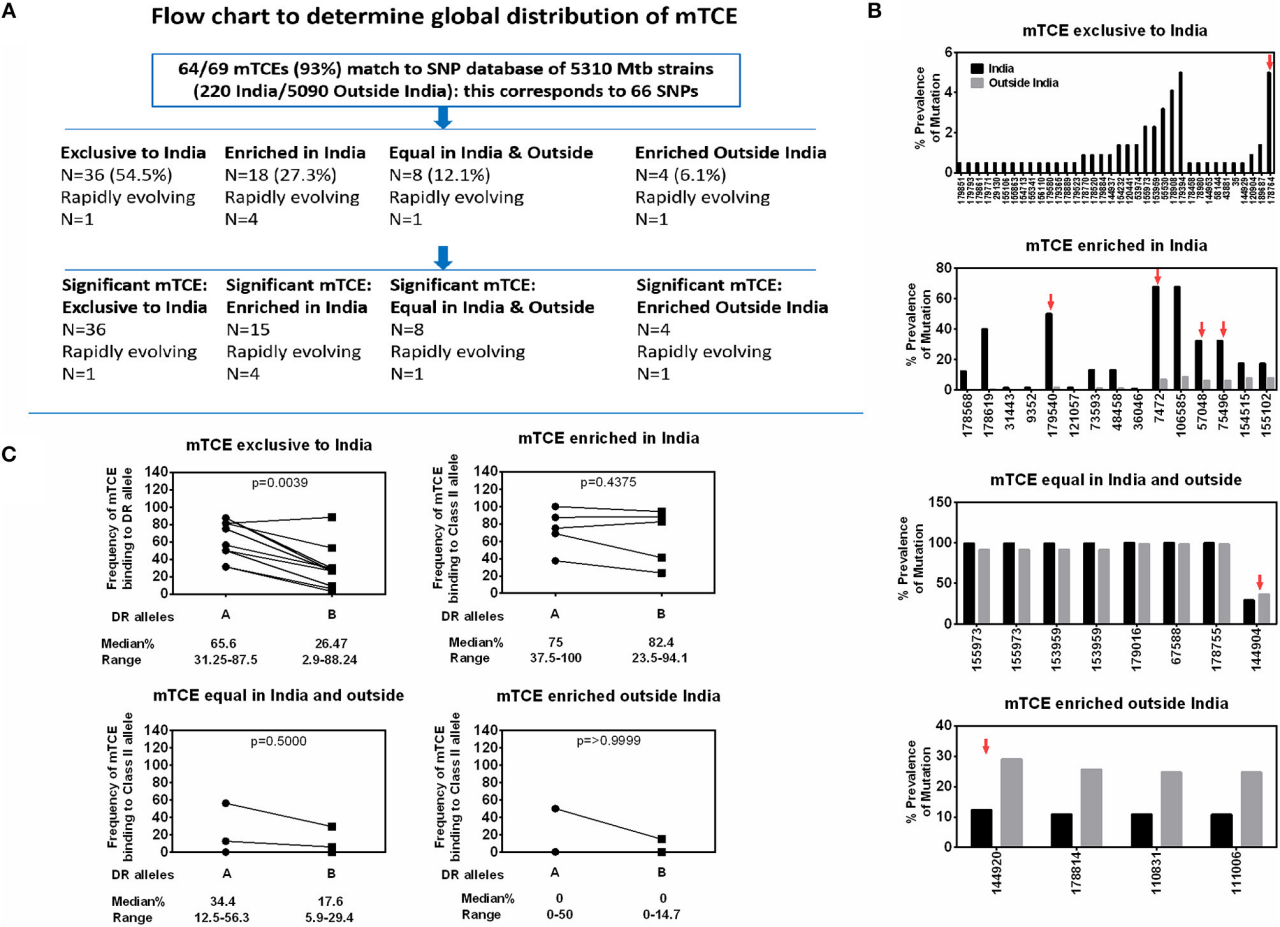
Global distribution of 64 mTCEs and relationship to HLA-DR binding. **(A)** Flow chart shows the number of mTCEs identified in 79 Mtb strains from India, and their frequency in the context of non-synonymous SNPs (nSNPs) identified in 5,310 globally distributed Mtb strains (18, 60). After excluding mTCEs from repetitive regions (*n* = 69/138), the remaining 69/138 mTCEs were mapped to nSNPs of 5,310 global Mtb strains, in which 64/69 mTCEs matched to corresponding 66 SNPs (some mTCEs have more than one mutation with each mutation corresponding to one SNP). These 66 SNPs in 64 mTCEs were classified into four groups based on their global geographic distribution in 5,310 Mtb strains as follows: mTCE exclusive to India; mTCE enriched in India; mTCE in equal proportion inside and outside India and mTCE enriched outside India. Of the 4 groups of mTCE identified, those that were significantly exclusive to (*N* = 36) enriched (*N* = 15) or enriched outside India (*N* = 4) as determined by Fischer's exact test adjusted for multiple comparison with a *p*-value cut-off of < 0.001 is shown. Only for one group: mTCE in equal proportion in both India and outside India (*N* = 8) all the mTCEs identified is shown irrespective of significance. **(B)** Each bar is an mTCE (IEDB Epitope ID of corresponding reference epitope) (X-axis list) that corresponds to the proportion of prevalence of mutation (Y Axis list) in India (black square) vs. outside India (gray square). Red arrow above the bar represents rapidly evolving epitopes. **(C)** Paired line graph show the frequency (percentage) of all 15-mer mTCE from each of the 4 groups listed in Figure 6B binding with high affinity (<10 nM) either to 16 HLA-DR alleles that are common in India (but also prevalent outside India) vs. a total of 34 HLA-DR alleles that are either not common in India or not reported in India. *P*-value determined by Wilcoxon matched-pairs signed rank *t*-test.

Screening for amino acid changes in the mTCEs identified against a SNP database of 5,310 Mtb whole genome sequences enabled the contextualization of T-cell epitope mutations in a large collection of global Mtb strains. Among 5,310 Mtb strains, 220 were from India (16), including the 79 we initially used to identify mTCE by the annotation-based and SNP-based methods (see Figure S1, Supplementary File 2, and Figure 6A for overview of analysis). The 64 mTCE matched 66 SNPs and identified four distinct clusters of Mtb strains: (i) mTCE exclusive to strains from India (*N* = 36/66 = 54.5%); (ii) mTCE enriched (and therefore more likely to be identified) in strains from India (*N* = 18/66 = 27.3%); (iii) mTCE in near equal proportion in strains from India and outside India (*N* = 8/66 = 12.1%) and (iv) mTCE enriched in strains outside India i.e., rare in India (*N* = 4/66 = 6.1%). The majority of mTCEs identified (82%) fell in groups (i) and (ii) above; they were either exclusive to Mtb strains circulating in India or occurred at a median 12.4-fold higher frequency in strains isolated from India compared to the rest of the world (range 2.2- to 156.2-fold higher) (Supplementary File 2; Sheet 3). Analysis of lineage distribution shows that mTCE accumulating in India largely include EAI and CAS strains and mTCE accumulating equally in India and outside India include the Beijing strains. A proportionality test adjusted for multiple comparisons identified mTCE that were significantly enriched in India vs. outside India and these are listed in “x” axis of Figure 6B. These data confirm the majority of T-cell epitope mutations reported in this study (82%) to occur significantly more frequently in strains circulating in India; only 6.1% of mTCEs identified were more prevalent in strains outside India (Figures 6A,B).

We next determined if this region-specific occurrence of mTCE was determined possibly by preferential binding to HLA Class-II alleles common in India vs. the rest of the world, given the recognized critical importance of HLA in driving immune selection. We hypothesized that the ten mTCEs (15-mer) that were exclusive to India (Figure 6B; Supplementary File 4) would bind preferentially to HLA Class-II alleles that were highly prevalent in India but rare in the rest of the world. To test this possibility, all 15-mer mTCEs of the 64 mTCE identified were probed for predicted binding to two major groups of HLA-DR alleles: Group A included 16 DR alleles that are highly prevalent in India, but also found outside India. Group B included 34 HLA-DR alleles that were either rare in India (*N* = 11) or absent in India (*N* = 23). To note: we did not identify any HLA-DR alleles exclusive to India, which were absent from the rest of the world. Figure 6C shows a Wilcoxon paired analysis comparing the percentage of Group A vs. Group B HLA-DR alleles, with predicted binding to each of the mTCE tested. In keeping with our prediction, the 10 mTCEs exclusive to India showed significantly higher predicted binding to HLA-DR alleles prevalent in India compared to DR alleles rare in India (*p* = 0.0039) whereas mTCEs that were enriched but not exclusive to India showed equally efficient predicted binding to both groups of DR alleles (*p* = 0.4375). All 15-mer mTCEs tested showed high affinity predicted binding to multiple DR alleles (>60%) prevalent in India (Figure 6C: refer to median percentage of each group of mTCEs binding to Group A DR alleles), with the exception of mTCEs equal in India and outside India and mTCEs enriched outside India, which showed predicted binding to only few (<35% median) DR alleles. Interestingly, HLA-DRB1^*^03, HLA-DRB1^*^04 & HLA-DRB1^*^08 have been associated with TB protection/disease (68, 69); Group-A includes HLA-DRB1^*^03:01, ^*^04:02, ^*^04:08, and ^*^04:10 all of which bind ≧60% (>12/20) peptides tested with high predicted binding affinity. Together, these data provide strong evidence that MHC Class II DR binding patterns likely reflect whether an mTCE is (i) exclusive to India, (ii) enriched in India but can be found infrequently globally, (iii) in equal proportion in India vs. the rest of the world or (iv) enriched outside India.

## Discussion

This study provides compelling evidence that (a) functional mutations in Mtb-specific CD4 T cell epitopes are not uncommon in Mtb strains isolated from infected subjects living in India—each of the 79 Mtb strains isolated from 79 infected subjects carried on average 7 mTCE (Figure 1, Figure S3); (b) the vast majority of SNPs in mTCEs that we report are region specific (Figure 6A) and either found exclusively in strains from India or to occur significantly more frequently in strains from India (Figure 6B); (c) 15.7% of mutated epitopes reported bear the hallmarks of emergence due to immune selection pressure with higher nucleotide diversity scores than the corresponding parent T cell epitope currently curated in the Immune Epitope Database (Figures 3, 4); and (d) *in silico* analysis shows altered binding of T cell epitopes to MHC Class-II HLA-DR alleles to be a potentially important mechanism contributing to the emergence of these region-specific T-cell epitope mutations in Mtb. Whilst the overall number of mutations in known T cell epitopes remains low, in keeping with previous reports highlighting TCE mutations to be rare in Mtb (13, 15), it is clear that all Mtb strains from India tested carry multiple mTCE (Figure S3), of which only seven have been previously been reported (13, 15). Sixty-two percent of mTCE tested have a functional consequence on CD4 T-cells IFNγ secretion, which is associated with predicted changes in MHC Class-II binding (Figure 5). Most importantly, the 64 mutations we report have been verified in an extensive database of SNP's generated on 5,310 global Mtb strains (46). Taken together, our study provides a comprehensive analysis of TCE mutations in Mtb and highlights an underappreciated level of region-specific TCE diversity accumulating in Mtb strains.

The remarkable diversity in T-cell epitope mutations in Mtb strains circulating worldwide vs. India highlights the underlying significance of regional differences in shaping their evolution in genetically (HLA) and environmentally (e.g., NTM) different regions. Despite analysis of a significant number of lineage-1-strains in the Coscolla et al. (15) study, very few (*n* = 3) of the mutations they report were found in our strains and vice versa. Although multiple strains isolated from a single donor over time reveal stability of mutations (Supplementary File 6), an analysis of the 79 strains isolated from India shows only 19% of the mutated epitopes to be common (Venn diagram, Figure S3), highlighting significant variation in T cell epitope mutations even within donors who map to a single geographical region. The fact that most mutated TCE reported in this study are lineage-specific highlights that changes in proteins that diverge between lineages can include proteins that encode TCEs. Our study also highlights that some lineage-specific TCEs may evolve due to immune selection pressure. Thus, 7 of the 64 total mTCEs that map to highly rapidly diversifying regions of the bacterial genome, which impact T-cell function and are present in multiple strains, are majorly found within the EAI lineage (Table 1). In addition, we identified five mTCEs that were common across three lineages and prevalent in ≧90% of strains studied; only one of these has been previously reported (13, 15). The top 10% of genes, in terms of nucleotide diversity, in Mtb strains from India encoded none of these five very common mutated epitopes. Therefore, the process that governs the selection of these highly common TCE mutations would be predicted to differ from those that are rapidly evolving and may be linked to more fundamental bacterial survival. Together, these observations indicate that diverse mechanisms may account for the unique patterns of T-cell epitope mutations accumulating in Mtb strains.

Previous studies (13, 15) have implied that immune escape from T-cell responses is unlikely to be a major factor contributing to Mtb diversity and persistence, on the basis that only a small percentage of known T cell epitopes that can induce an immune response are mutated, despite clear evidence that the bacterial genome itself is evolving. Whilst our study concurs with this notion (13, 15), this observation needs to be placed in context of data that suggest that current studies underestimate the number of TCEs in Mtb. For example, Coscolla et al. (15) recently highlighted the presence of several new TCE, not curated previously in the IEDB, encoded by Mtb proteins that are rapidly diversifying, which carry functional mutations. This, along with our data emphasizes the importance of probing highly diverging genes of Mtb for TCE mutations. Indeed, the analysis of complete genome sequences from clinical isolates has identified the Mtb genome to carry SNPs, large sequence polymorphisms (LSPs), and regions of difference (RDs) originating from small deletions, deletions in homologous repetitive elements, point mutations, genome rearrangements and frame-shift mutations (70). The significance of this inherent genetic variation is not fully understood, but has been suggested to denote selective pressure and therefore be important in bacterial pathogenesis and immunity (70, 71). In our study, given that screening the large global Mtb genome SNP database of 5,310 strains confirmed the mutated epitopes identified to be largely region-specific, we did not apply selection analysis as calculating the non-synonymous vs. synonymous substitution changes (dn/ds) are not deemed appropriate for strain level analysis within a species, though suitable for inter-species level comparisons (72).

These observations of Mtb diversity in the context of our data call for further functional studies on Mtb-specific TCE changes to better understand their impact on immune escape and bacterial persistence. In addition, studies are needed to verify epitopes predicted within highly divergent regions. One limitation of our analysis is known difficulties in interpreting sequencing data from repetitive regions of the genome, particularly when data are generated from a single sequencing library of short genomic fragments as in this study. For instance, ESAT-6 encoding regions are known to be difficult to sequence, assemble and align using short read technologies causing errors that could inflate nucleotide diversity estimates. However, previous works using similar data have reported on TCE mutations in these same genes (13, 15) and we also elected to follow up only on those variants appearing in both assembly and variant detection analyses thus minimizing this issue. In this case, only one epitope identified in a repetitive gene ESAT-6 was retained, which showed 2 variants, especially as this particular mutation was previously reported to have a functional consequence.

The discovery that immune selection pressure can potentially drive TCE mutations in Mtb and our data that emerging TCEs differ markedly between Mtb strains circulating worldwide vs. in India, has implications for vaccine design. Firstly, our data and those of Coscolla et al. (15), pave the way for these new epitopes with functional impact in proteins that are rapidly diversifying to be validated for their ability to detect T-cells that correlate with protection. If validated, this will greatly improve vaccine design by enabling focused efforts on vaccines designed to induce T-cells with such specificities. Further, the accumulating evidence that the T-cell epitope landscape is likely globally diverse and evolving, emphasizes the need to factor this information in future vaccine design. Taken together, our data therefore provides novel insight to a key, understudied aspect of TB immunity: namely, the nature and pattern of CD4 T-cell epitope mutations accumulating in Mtb genomes.

## Ethics Statement

A total of 130 individuals were prospectively recruited between April 2015 and April 2016 at St. John's Medical College and Hospital and the study was approved by the Ethical Review Committee of St. John's Medical College Hospital, Bangalore, India (Ref no: 55/2015). Subjects were included in this study only after obtaining written consent. Relevant clinical information was documented in a pro forma. All adult subjects provided informed consent, and a parent or guardian of any child participant provided informed consent on the child's behalf.

## Author Contributions

AR and AV conceived the project. AR, SR, and AV designed the experiments. AR and SoN performed the main bioinformatic and immuno-informatic analyses. SR and PS performed the immunology experiments. AM, TA, and AE sequenced the genomes and conducted SNP and phylogenetic analyses. SiS, AJ, JS, SuN, GD, and SoS collected clinical samples, provided the patient details and wrote the clinical methodology for the manuscript. PvH synthesized the peptides. TO contributed in immunology study design. AR, SR, and AV wrote the manuscript. AR, SR, AM, AE, TA, TO, and AV edited the manuscript.

### Conflict of Interest Statement

The authors declare that the research was conducted in the absence of any commercial or financial relationships that could be construed as a potential conflict of interest.

## Supplementary Material

Figure S1 Pipeline for TCEs screening. Flow chart shows the number of T-cell epitopes retrieved from IEDB, number of TCEs identical to reference Mtb strain H37Rv and their corresponding antigens, and number of TCEs finally considered for further analyses.

Figure S2 Pie chart representing functional categories of mTCEs and reference/parent TCE. **(A)** 64 mTCEs; **(B)** Parent 905 TCEs.

Figure S3 mTCEs by clinical classification. All 79 strains were classified into five clinical groups. Strains in each of 5 groups are further classified into 3 subsets: good, bad and default, based on their clinical outcomes. Stack bars represented a total number of mTCEs in each of 79 strains studied. **(A)** 26 drug sensitive (DS) HIV-negative strains, **(B)** 22 DS HIV-positive strains, **(C)** 12 poly-resistant (PR) HIV-negative strains, **(D)** 15 multi drug-resistant (MDR) HIV-negative strains, **(E)** 4 MDR HIV-positive strains, and **(F)** Venn diagram based on 53 mTCEs sequences from 5 different clinical categories, showing mTCEs that are common and unique to each category. Only epitopes that are annotated in all 79 Mtb strains were considered for this analysis, after excluding epitopes encoded by partially annotated antigens in some strains. Clinical outcomes—Good, patient successfully treated; Bad, patient not cured of TB; Default, patient relapsed during treatment.

Figure S4 Novel epitopes induce a stronger IFNγ response in IGRA^+^ individuals, but response to immunodominant antigens are similar in both clinical groups. **(A)** Diluted heparinized whole blood from 12 IGRA^+^ individuals and 6 PTB patients were stimulated with a total of 36 parent and corresponding mutant peptides at 1 μg/ml final concentration for 7 days. Summarized data shows the proportion of responders in the 7-day whole blood IFNγ release assay to a total of 36 parent (blue) and corresponding mutant (red) peptides in IGRA^+^ and PTB subjects, with a cut-off for a positive IFNγ response set at 5 pg/ml. Peptides that induced responses in > = 25% of donors tested are indicated with a horizontal black line. Nine peptides marked by pink arrows were also simultaneously tested in ICS assay as shown in Figure 4. Thirteen 15 mer peptides representing parent and mutant sequences of epitopes marked by green asterisks were selected for HLA binding assay as shown in Figure 5 and whose IFNγ response is illustrated in Figure S6. **(B)** Diluted heparinized whole blood from 10 healthy IGRA^−^ individuals, 10 latent TB-infected (IGRA^+^) and 10 pulmonary TB (PTB) were either left unstimulated or stimulated with Mtb antigens ESAT6/CFP10 and PPD at 10 μg/ml and positive control PHA (3 μg/ml) for 7 days. Scatter plot shows IFNγ levels in the supernatants measured by ELISA after background unstimulated control value subtraction. Each dot represents a donor. Data was analyzed using Non-parametric One-Way ANOVA Kruskal-Wallis test and Dunn's multiple comparisons test. Significant differences are indicated: ^**^*p* < 0.01.

Figure S5 mTCEs induce a highly specific CD4 T cell response in IGRA^+^ subjects. **(A)** PBMCs from IGRA^+^ and IGRA^−^ subjects were stimulated overnight with rapidly evolving immunodominant parent (P3-3, upper panel) and mutant peptide (M4-3, lower panel). Representative flow cytometry plots depicting IFNγ^+^ and IL2^+^ CD4 T cells upon stimulation of PBMCs in IGRA^+^ (left) and IGRA^−^ (right) is shown. **(B)** IFNγ and/or IL2 CD4 T cell frequency following stimulation with a set of five parent and mutant peptides in six IGRA^−^ subjects after subtraction of background-unstimulated control. The mean/SD response frequency in the six IGRA^−^ donors to each of the five parent and mutant peptides tested is shown as five lines (one line per peptide pair). Median responses in IGRA^−^ subjects to the peptides tested, after background subtraction, was: parent peptide median 0.02, range 0–0.175; mutant median 0.0365, range 0–0.165. These data confirm these peptides to have negligible non-specific stimulatory effects in this assay.

Figure S6 Whole blood IFNγ levels in response to 13 15 mer synthetic peptides representing parent and mutant sequences of epitopes selected for HLA binding assay. IFNγ ELISA was performed on supernatants collected after 7-day stimulation of diluted heparinized blood from IGRA^+^ and PTB subjects with peptides. Each graph shows the concentration of IFNγ (pg/ml), which either induces gain **(A)** or loss **(B)** or no change in function **(C)**. Each line represents IFN**γ** levels induced by a given donor in response to the parent vs. the mutant peptide assayed simultaneously after subtraction of background-unstimulated control. Statistical analysis was performed by Paired Wilcoxon signed-rank test.

Figure S7 CD4 T cell responses to parent and mutant peptides. PBMCs from IGRA^+^ subjects were stimulated overnight with parent and mutant peptides that are rapidly evolving and belonging to immunodominant and non-immunodominant category. Frequencies of IFNγ^+^, IL2^+^, TNFα^+^, IL17A^+^, and MIP1β^+^ CD4 T cells were determined by ICS assay (see methods). Line graphs shows frequencies of cytokine-positive CD4 T cells following stimulation with a set of six parent and mutant peptides as individual plots in IGRA^+^ subjects after subtraction of background unstimulated control. Each line is a donor. Statistical analysis was performed using nonparametric Wilcoxon matched-pairs signed rank test. ^*^*P* < 0.05 was considered significant. mTCE were grouped as follows: **(A)** Rapidly evolving and immunodominant Rv3019c (EsxR) and Rv0294 (tam) **(B)** Rapidly evolving and non-immunodominant Rv0988, Rv3879c (espK) and Rv1769.

Supplementary File 1 Frequency and length of T-cell epitopes analyzed. **(A)** 905 parent/reference TCEs, **(B)** 64 mTCEs.

Supplementary File 2 Comprehensive data on 64 mTCEs identified and their frequency in 5,310 globally distributed Mtb strains (18, 60). Sheet 1 contains the total number of mTCEs (*n* = 138) that we initially identified in both non-repetitive and repetitive regions of the 79 Mtb strains we studied, along with complete relevant data. Sheet 2 contains the identical information of Sheet 1, but excluding mTCEs (*n* = 69) from repetitive regions. Sheet 3 shows the matching non-synonymous SNPs (nSNPs) from 5,310 globally distributed Mtb strains with the mutations of mTCEs identified from non-repetitive regions of the 79 Mtb strains we studied. In total, 64 mTCEs have a match in 5,310 global strains, but as some of the mTCEs have more than one mutation, the total number of SNPs recorded is 66. Sheet 4 shows only those mTCEs from Sheet 3 that are rapidly evolving. Sheet 5 shows all relevant information about the epitopes having multiple forms.

Supplementary File 3 Nucleotide diversity of all the genes from 79 Mtb isolates we studied from India.

Supplementary File 4 HLA binding affinity prediction of all 15 mer parent/reference TCE and their mutants (*N* = 20) to total of 50 MHC Class-II alleles (http://allelefrequencies.net/). Allele frequency ≥0.01 is set-up as cut-off to screen high frequency alleles. The 20 mTCEs are classified into four groups based on their global geographic distribution in 5,310 Mtb strains as follows: mTCE exclusive to India; mTCE enriched in India; mTCE in equal proportion inside and outside India and mTCE enriched outside India. Total of 50 alleles are classified as follows: Binding of each mTCE to 16 DR alleles (Group A) common in India (but also prevalent outside India) was compared to 34 DR alleles (Group B) comprising 11 that are less prevalent in India but highly enriched outside India and 23 alleles that are not reported to date in India, but prevalent in rest of the world (geographical regions): Australia, Europe, North Africa, North America, North-East Asia, Oceania, South and Central America, South-East Asia, Sub-Saharan Africa and Western Asia. These 23 alleles are reported with high frequency in at-least two of 10 geographical regions we considered. Predicted binding affinity of all four groups of TCE/mTCEs are provided against all alleles we studied.

Supplementary File 5 Clinical details of samples used for testing T-cell function of mutant vs. parent peptides.

Supplementary File 6 Details of intra-donor Mtb T-cell epitope mutations in six subjects. A total of 17 Mtb strains (range 2–5 strains) were isolated from 6 subjects (Patient ID: P6, P38, P96, P97, P103, and P106). The total number of Mtb strains isolated from each donor, along with number of mTCEs and mutated antigens identified in strains isolated over multiple time points is summarized in individual sheets of EXCEL this File for each of six donors tested.

Table S1 Details of 5 mTCE identified in ≥90% of 79 Mtb strains from India.

Table S2 Clinical details of individuals included in this study.

## References

[1] Cobey S. Pathogen evolution and the immunological niche. Ann N Y Acad Sci. (2014) 1320:1–15. 10.1111/nyas.12493 25040161 PMC4141700

[2] Brites D Gagneux S. Old and new selective pressures on *Mycobacterium tuberculosis*. Infect Genet Evol. (2012) 12:678–685. 10.1016/j.meegid.2011.08.010 21867778 PMC3253320

[3] Pepperell CS Casto AM Kitchen A Grana JM Cornejo OE Holmes EC The role of selection in shaping diversity of natural *Mycobacterium tuberculosis* populations. PLoS Pathog. (2013) 9:e1003543 10.1371/journal.ppat.1003543 23966858 PMC3744410

[4] Kwan CK Ernst JD. HIV and tuberculosis: a deadly human syndemic. Clin Microbiol Rev. (2011) 24:351–76. 10.1128/CMR.00042-10 21482729 PMC3122491

[5] Lin MY Reddy TB Arend SM Friggen AH Franken KL van Meijgaarden KE. Cross-reactive immunity to *Mycobacterium tuberculosis* DosR regulon-encoded antigens in individuals infected with environmental, nontuberculous mycobacteria. Infect Immun. (2009) 77:5071–9. 10.1128/IAI.00457-09 19737909 PMC2772522

[6] North RJ Jung YJ. Immunity to tuberculosis. Annu Rev Immunol. (2004) 22:599–623. 10.1146/annurev.immunol.22.012703.104635 15032590

[7] Kara EE Comerford I Fenix KA Bastow CR Gregor CE McKenzie DR. Tailored immune responses: novel effector helper T cell subsets in protective immunity. PLoS Pathog. (2014) 10:e1003905. 10.1371/journal.ppat.1003905 24586147 PMC3930558

[8] Black GF Thiel BA Ota MO Parida SK Adegbola R Boom WH. Immunogenicity of novel DosR regulon-encoded candidate antigens of *Mycobacterium tuberculosis* in three high-burden populations in Africa. Clin Vaccine Immunol. (2009) 16:1203–12. 10.1128/CVI.00111-09 19553548 PMC2725533

[9] Commandeur S van Meijgaarden KE Prins C Pichugin AV Dijkman K van den Eeden SJ. An unbiased genome-wide *Mycobacterium tuberculosis* gene expression approach to discover antigens targeted by human T cells expressed during pulmonary infection. J Immunol. (2013) 190:1659–71. 10.4049/jimmunol.1201593 23319735

[10] Bhatt K Verma S Ellner JJ Salgame P. Quest for correlates of protection against tuberculosis. Clin Vaccine Immunol. (2015) 22:258–66. 10.1128/CVI.00721-14 25589549 PMC4340894

[11] Lancioni C Nyendak M Kiguli S Zalwango S Mori T Mayanja-Kizza H. CD8+ T cells provide an immunologic signature of tuberculosis in young children. Am J Respir Crit Care Med. (2012) 185:206–12. 10.1164/rccm.201107-1355OC 22071329 PMC3297089

[12] Ernst JD. The immunological life cycle of tuberculosis. Nat Rev Immunol. (2012) 12:581–91. 10.1038/nri3259 22790178

[13] Comas I Chakravarthi J Small PM Galagan J Niemann S Kremer K. Human T cell epitopes of *Mycobacterium tuberculosis* are evolutionarily hyperconserved. Nature Genet. (2010) 42:498–503. 10.1038/ng.590 20495566 PMC2883744

[14] Vita R Overton JA Greenbaum JA Ponomarenko J Clark JD Cantrell JR. The immune epitope database (IEDB) 3.0. Nucl Acids Res. (2015) 43:D405–12. 10.1093/nar/gku938 25300482 PMC4384014

[15] Coscolla M Copin R Sutherland J Gehre F de Jong B Owolabi O. *M. tuberculosis* T cell epitope analysis reveals paucity of antigenic variation and identifies rare variable TB antigens. Cell Host Microbe. (2015) 18:538–48. 10.1016/j.chom.2015.10.008 26607161 PMC4758912

[16] Manson AL Cohen KA Abeel T Desjardins CA Armstrong DT Barry CE. Genomic analysis of globally diverse *Mycobacterium tuberculosis* strains provides insights into the emergence and spread of multidrug resistance. Nat Genet. (2017) 49:395–402. 10.1038/ng.3767 28092681 PMC5402762

[17] Manson AL Abeel T Galagan JE Sundaramurthi JC Salazar A Gehrmann T. *Mycobacterium tuberculosis* whole genome sequences from Southern India suggest novel resistance mechanisms and the need for region-specific diagnostics. Clin Infect Dis. (2017) 64:1494–501. 10.1093/cid/cix169 28498943 PMC5434337

[18] Bronke C Almeida CA McKinnon E Roberts SG Keane NM Chopra A. HIV escape mutations occur preferentially at HLA-binding sites of CD8 T-cell epitopes. AIDS (2013) 27:899–905. 10.1097/QAD.0b013e32835e1616 23276808 PMC3818524

[19] Ellis RC Zabrowarny LA. Safer staining method for acid fast bacilli. J Clin Pathol. (1993) 46:559–60. 10.1136/jcp.46.6.559 7687254 PMC501296

[20] Allen BW Baker FJ Mycobacteria: Isolation, Identification and Sensitivity Testing (Laboratory Aids), (London: Butterworth-Heinemann) (1968) p. 1–75.

[21] Canetti G Fox W Khomenko A Mahler HT Menon NK Mitchison DA. Advances in techniques of testing Mycobacterial drug sensitivity, and the use of sensitivity tests in tuberculosis control programmes. Bull World Health Organ. (1969) 41:21–43. 5309084 PMC2427409

[22] Baess I. Isolation and purification of deoxyribonucleic acid from Mycobacteria. Acta Pathol Microbiol Scand B Microbiol Immunol. (1974) 82:780–4. 10.1111/j.1699-0463.1974.tb02375.x 4617483

[23] Kamerbeek J Schouls L Kolk A van Agterveld M van Soolingen D Kuijper S. Simultaneous detection and strain differentiation of *Mycobacterium tuberculosis* for diagnosis and epidemiology. J Clin Microbiol. (1997) 35:907–14. 9157152 10.1128/jcm.35.4.907-914.1997 PMC229700

[24] Winglee K Manson McGuire A Maiga M Abeel T Shea T Desjardins CA. Whole Genome Sequencing of *Mycobacterium africanum* strains from Mali provides insights into the mechanisms of geographic restriction. PLoS Negl Trop Dis. (2016) 10:e0004332. 10.1371/journal.pntd.0004332 26751217 PMC4713829

[25] Cohen KA Abeel T Manson McGuire A Desjardins CA Munsamy V Shea TP. Evolution of extensively drug-resistant tuberculosis over four decades: whole genome sequencing and dating analysis of *Mycobacterium tuberculosis* isolates from KwaZulu-Natal. PLoS Med. (2015) 12:e1001880. 10.1371/journal.pmed.1001880 26418737 PMC4587932

[26] Kurtz S Phillippy A Delcher AL Smoot M Shumway M Antonescu C. Versatile and open software for comparing large genomes. Genome Biol. (2004) 5:R12. 10.1186/gb-2004-5-2-r12 14759262 PMC395750

[27] Hyatt D Chen GL Locascio PF Land ML Larimer FW Hauser LJ. Prodigal: prokaryotic gene recognition and translation initiation site identification. BMC Bioinform. (2010) 11:119. 10.1186/1471-2105-11-119 20211023 PMC2848648

[28] Lowe TM Eddy SR. tRNAscan-SE: a program for improved detection of transfer RNA genes in genomic sequence. Nucl Acids Res. (1997) 25:955–64. 10.1093/nar/25.5.955 9023104 PMC146525

[29] Lagesen K Hallin P Rodland EA Staerfeldt HH Rognes T Ussery DW. RNAmmer: consistent and rapid annotation of ribosomal RNA genes. Nucl Acids Res. (2007) 35:3100–8. 10.1093/nar/gkm160 17452365 PMC1888812

[30] Finn RD Bateman A Clements J Coggill P Eberhardt RY Eddy SR. The Pfam protein families database. Nucl Acids Res. (2014) 42:D222–30. 10.1093/nar/gkt1223 24288371 PMC3965110

[31] Haft DH Loftus BJ Richardson DL Yang F Eisen JA Paulsen IT. TIGRFAMs: a protein family resource for the functional identification of proteins. Nucl Acids Res. (2001) 29:41–3. 10.1093/nar/29.1.41 11125044 PMC29844

[32] Ogata H Goto S Sato K Fujibuchi W Bono H Kanehisa M. KEGG: Kyoto Encyclopedia of genes and genomes. Nucl Acids Res. (1999) 27:29–34. 10.1093/nar/27.1.29 9847135 PMC148090

[33] Tatusov RL Koonin EV Lipman DJ. A genomic perspective on protein families. Science (1997) 278:631–7. 10.1126/science.278.5338.631 9381173

[34] Conesa A Gotz S Garcia-Gomez JM Terol J Talon M Robles M. Blast2GO: a universal tool for annotation, visualization and analysis in functional genomics research. Bioinformatics (2005) 21:3674–76. 10.1093/bioinformatics/bti610 16081474

[35] Tian W Arakaki AK Skolnick J. EFICAz: a comprehensive approach for accurate genome-scale enzyme function inference. Nucl Acids Res. (2004) 32:6226–39. 10.1093/nar/gkh956 15576349 PMC535665

[36] Petersen TN Brunak S von Heijne G Nielsen H. SignalP 4.0: discriminating signal peptides from transmembrane regions. Nat Methods (2011) 8:785–6. 10.1038/nmeth.1701 21959131

[37] Krogh A Larsson B von Heijne G Sonnhammer EL. Predicting transmembrane protein topology with a hidden Markov model: application to complete genomes. J Mol Biol. (2001) 305:567–80. 10.1006/jmbi.2000.4315 11152613

[38] Wapinski I Pfeffer A Friedman N Regev A. Natural history and evolutionary principles of gene duplication in fungi. Nature (2007) 449:54–61. 10.1038/nature06107 17805289

[39] Wapinski I Pfeffer A Friedman N Regev A. Automatic genome-wide reconstruction of phylogenetic gene trees. Bioinformatics (2007) 23:i549–58. 10.1093/bioinformatics/btm193 17646342

[40] Li H Durbin R. Fast and accurate short read alignment with Burrows-Wheeler transform. Bioinformatics (2009) 25:1754–60. 10.1093/bioinformatics/btp324 19451168 PMC2705234

[41] Walker BJ Abeel T Shea T Priest M Abouelliel A Sakthikumar S. Pilon: An integrated tool for comprehensive microbial variant detection and genome assembly improvement. PLoS ONE (2014) 9:e112963. 10.1371/journal.pone.0112963 25409509 PMC4237348

[42] Stamatakis A. RAxML-VI-HPC: Maximum likelihood-based phylogenetic analyses with thousands of taxa and mixed models. Bioinformatics (2006) 22:2688–690. 10.1093/bioinformatics/btl446 16928733

[43] Abeel T Phylogenetic Visualizations With Peacock. (2016). Available online at: https://github.com/AbeelLab/peacock

[44] Haas B VCFAnnotator. (2012). Available online at: http://sourceforge.net/projects/vcfannotator/

[45] Lew JM Kapopoulou A Jones LM Cole ST. TubercuList-−10 years after. Tuberculosis (2011) 91:1–7. 10.1016/j.tube.2010.09.008 20980199

[46] Benjamini Y Hochberg Y Controlling the false discovery rate: a practical and powerful approach to multiple testing. J R Stat Soc Series B. (1995) 57:289–300. 10.1111/j.2517-6161.1995.tb02031.x

[47] Nielsen M Lundegaard C Worning P Lauemoller SL Lamberth K Buus S Reliable prediction of T-cell epitopes using neural networks with novel sequence representation. Protein Sci. (2003) 12:1007–17. 10.1110/ps.0239403 12717023 PMC2323871

[48] Lundegaard C Lamberth K Harndahl M Buus S Lund O Nielsen M. NetMHC-3.0: Accurate web accessible predictions of human, mouse and monkey MHC class i affinities for peptides of length 8-11. Nucl Acids Res. (2008) 36:W509–12. 10.1093/nar/gkn202 18463140 PMC2447772

[49] Wang P Sidney J Dow C Mothe B Sette A Peters B. A systematic assessment of MHC class II peptide binding predictions and evaluation of a consensus approach. PLoS Comput Biol. (2008) 4:e1000048. 10.1371/journal.pcbi.1000048 18389056 PMC2267221

[50] Wang P Sidney J Kim Y Sette A Lund O Nielsen M. Peptide binding predictions for HLA DR, DP and DQ molecules. BMC Bioinform. (2010) 11:568. 10.1186/1471-2105-11-568 21092157 PMC2998531

[51] Gonzalez-Galarza FF Christmas S Middleton D Jones AR. Allele frequency net: a database and online repository for immune gene frequencies in worldwide populations. Nucl Acids Res. (2011) 39:D913–19. 10.1093/nar/gkq1128 21062830 PMC3013710

[52] Greenbaum J Sidney J Chung J Brander C Peters B Sette A. Functional classification of class II human leukocyte antigen (HLA) molecules reveals seven different supertypes and a surprising degree of repertoire sharing across supertypes. Immunogenetics (2011) 63:325–35. 10.1007/s00251-011-0513-0 21305276 PMC3626422

[53] Weiskopf D Angelo MA de Azeredo EL Sidney J Greenbaum JA Fernando AN. Comprehensive analysis of dengue virus-specific responses supports an HLA-linked protective role for CD8+ T cells. Proc Natl Acad Sci USA. (2013) 110:E2046–53. 10.1073/pnas.1305227110 23580623 PMC3670335

[54] Pattnaik S John KR Shalini E Michael JS. Agreement between skin testing and QuantiFERON-TB Gold In-Tube assay (QFT-TB) in detecting latent tuberculosis infection among household contacts in India. Indian J Tuberc. (2012) 59:214–8. 23342541

[55] Franken KL Hiemstra HS van meijgaarden KE Subronto Y den hartigh J Ottenhoff TH. Purification of his-tagged proteins by immobilized chelate affinity chromatography: the benefits from the use of organic solvent. Protein Expr Purif. (2000) 18:95–9. 10.1006/prep.1999.1162 10648174

[56] Serra-Vidal MM Latorre I Franken KL Diaz J de Souza-Galvao ML Casas I. Immunogenicity of 60 novel latency-related antigens of *Mycobacterium tuberculosis*. Front Microbiol. (2014) 5:517. 10.3389/fmicb.2014.00517 25339944 PMC4189613

[57] Smith SG Smits K Joosten SA van Maijgaarden KE Satti I Fletcher HA TBVI TB Biomarker working group. intracellular cytokine staining and flow cytometry: considerations for application in clinical trials of novel tuberculosis vaccines. PLoS ONE (2015) 10:e0138042 10.1371/journal.pone.0138042 26367374 PMC4569436

[58] Lindestam Arlehamn CS Gerasimova A Mele F Henderson R Swann J Greenbaum JA Memory T cells in latent *Mycobacterium tuberculosis* infection are directed against three antigenic islands and largely contained in a CXCR3^+^CCR6^+^ Th1 subset. PLoS Pathog. (2013) 9:e1003130 10.1371/journal.ppat.1003130 23358848 PMC3554618

[59] Nei M Li WH. Mathematical model for studying genetic variation in terms of restriction endonucleases. Proc Natl Acad Sci USA. (1979) 76:5269–73. 10.1073/pnas.76.10.5269 291943 PMC413122

[60] Commandeur S Lin MY van Meijgaarden KE Friggen AH Franken KL Drijfhout JW. Double- and monofunctional CD4? and CD8? T-cell responses to *Mycobacterium tuberculosis* DosR antigens and peptides in long-term latently infected individuals. Eur J Immunol. (2011) 41:2925–36. 10.1002/eji.201141602 21728172

[61] Leyten EMS Lin MY Franken KL Friggen AH Prins C van Maijgaarden KE. Human T-cell responses to 25 novel antigens encoded by genes of the dormancy regulon of *Mycobacterium tuberculosis*. Microb Infect. (2006) 8:2052–60. 10.1016/j.micinf.2006.03.018 16931093

[62] Schuck SD Mueller H Kunitz F Neher A Hoffmann H Franken KLCM T cell responses against subdominant antigens indicate latent *Mycobacterium tuberculosis* infection. PLoS ONE (2009) 4:e5590 10.1371/journal.pone.0005590 19440342 PMC2680040

[63] Goletti D Butera O Vanini V Lauria FN Lange C Franken KL. Response to Rv2628 latency antigen associates with cured tuberculosis and remote infection. Eur Respir J. (2009) 36:135–42. 10.1183/09031936.00140009 19926735

[64] Rakshit S Adiga V Nayak S Sahoo PN Sharma PK van Meijgaarden KE. Circulating *Mycobacterium tuberculosis* DosR latency antigen-specific, polyfunctional, regulatory IL10(+) Th17 CD4 T-cells differentiate latent from active tuberculosis. Sci Rep. (2017) 7:11948. 10.1038/s41598-017-10773-5 28931830 PMC5607261

[65] Harris DP Vordermeier HM Arya A Moreno C Ivanyi J. Permissive recognition of a mycobacterial T-cell epitope: localization of overlapping epitope core sequences recognized in association with multiple major histocompatibility complex class II I-A molecules. Immunol (1995) 84:555–61. 7790029 PMC1415164

[66] Ivanyi J. Function and Potentials of M. tuberculosis Epitopes. Front Immunol. (2014) 5:107. 10.3389/fimmu.2014.00107 24715888 PMC3970012

[67] Ivanyi J Ottenhoff TH. Significance of antigen and epitope specificity in tuberculosis. Front Immunol. (2014) 5:524. 10.3389/fimmu.2014.00524 25379038 PMC4206936

[68] Yang PL He XJ Zang QJ Li HP Wang GY Qin J. Association of human leukocyte antigen DRB1 polymorphism and tuberculosis: a meta-analysis. Int J Tuberc Lung Dis. (2016) 20:121–8. 10.5588/ijtld.14.0930 26688538

[69] Lindestam Arlehamn CS McKinney DM Carpenter C Paul S Rozot V Makgotlho E A Quantitative analysis of complexity of human pathogen-specific CD4 TCell Responses in Healthy *M. tuberculosis* infected South Africans. PLoS Pathog. (2016) 12:e1005760 10.1371/journal.ppat.1005760 27409590 PMC4943605

[70] Fleischmann RD Alland D Disen JA Carpenter L White O Peterson J. Whole-genome comparison of *Mycobacterium tuberculosis* clinical and laboratory strains. J Bacteriol. (2002) 184:5479–90. 10.1128/JB.184.19.5479-5490.2002 12218036 PMC135346

[71] Gagneux S DeRiemer K Van T Kato-Maeda M de Jong BC Narayanan S. Variable host-pathogen compatibility in *Mycobacterium tuberculosis*. Proc Natl Acad Sci USA. (2006) 103:2869–73. 10.1073/pnas.0511240103 16477032 PMC1413851

[72] Kryazhimskiy S Plotkin JB. The population genetics of dN/dS. PLoS Genet. (2008) 4:e1000304. 10.1371/journal.pgen.1000304 19081788 PMC2596312

