# Supplementary material for: Evidence for Highly Variable, Region-Specific Patterns of T-Cell Epitope Mutations Accumulating in Mycobacterium tuberculosis Strains
Source: Front Immunol. 2019 Feb 13;10:195. doi: 10.3389/fimmu.2019.00195 (PMC6381025; doi:10.3389/fimmu.2019.00195)
Supplement: Figure S1 — Pipeline for TCEs screening. Flow chart shows the number of T-cell epitopes retrieved from IEDB, number of TCEs identical to reference Mtb strain H37Rv and their corresponding antigens, and number of TCEs finally considered for further analyses. [file Presentation_1.PPTX]

## Slide 1
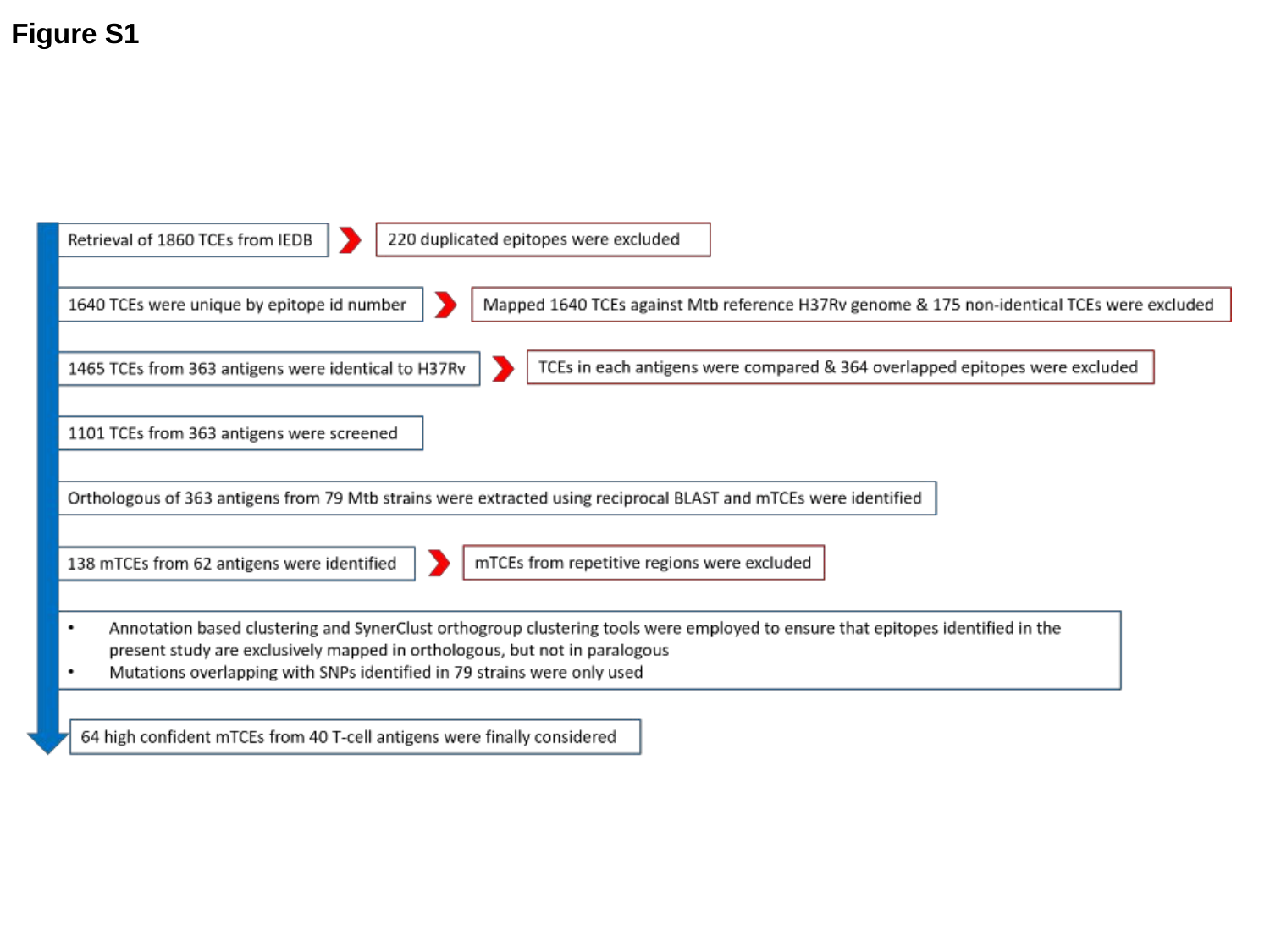

Figure S1

## Slide 2
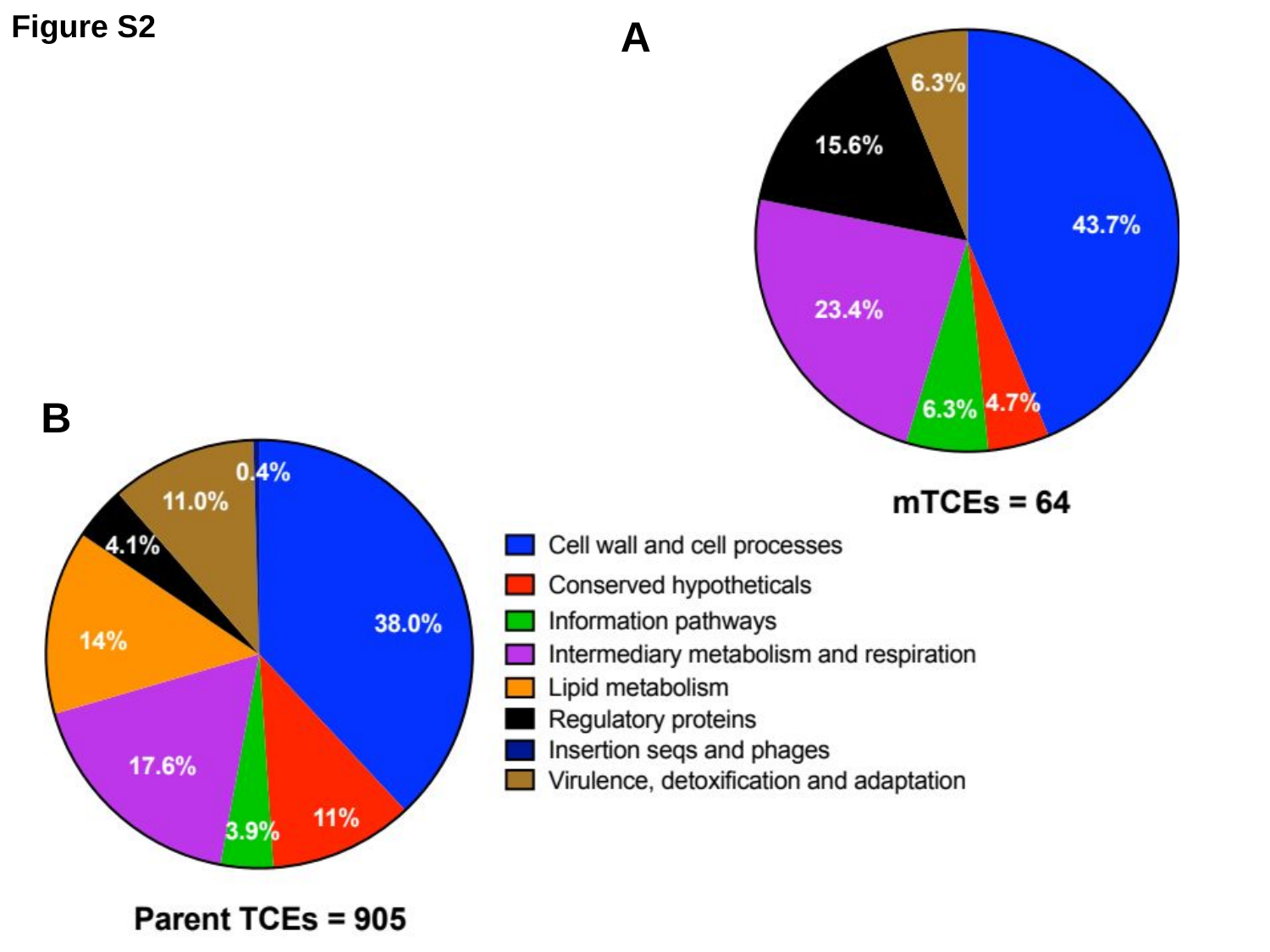

Figure S2
A
B

## Slide 3
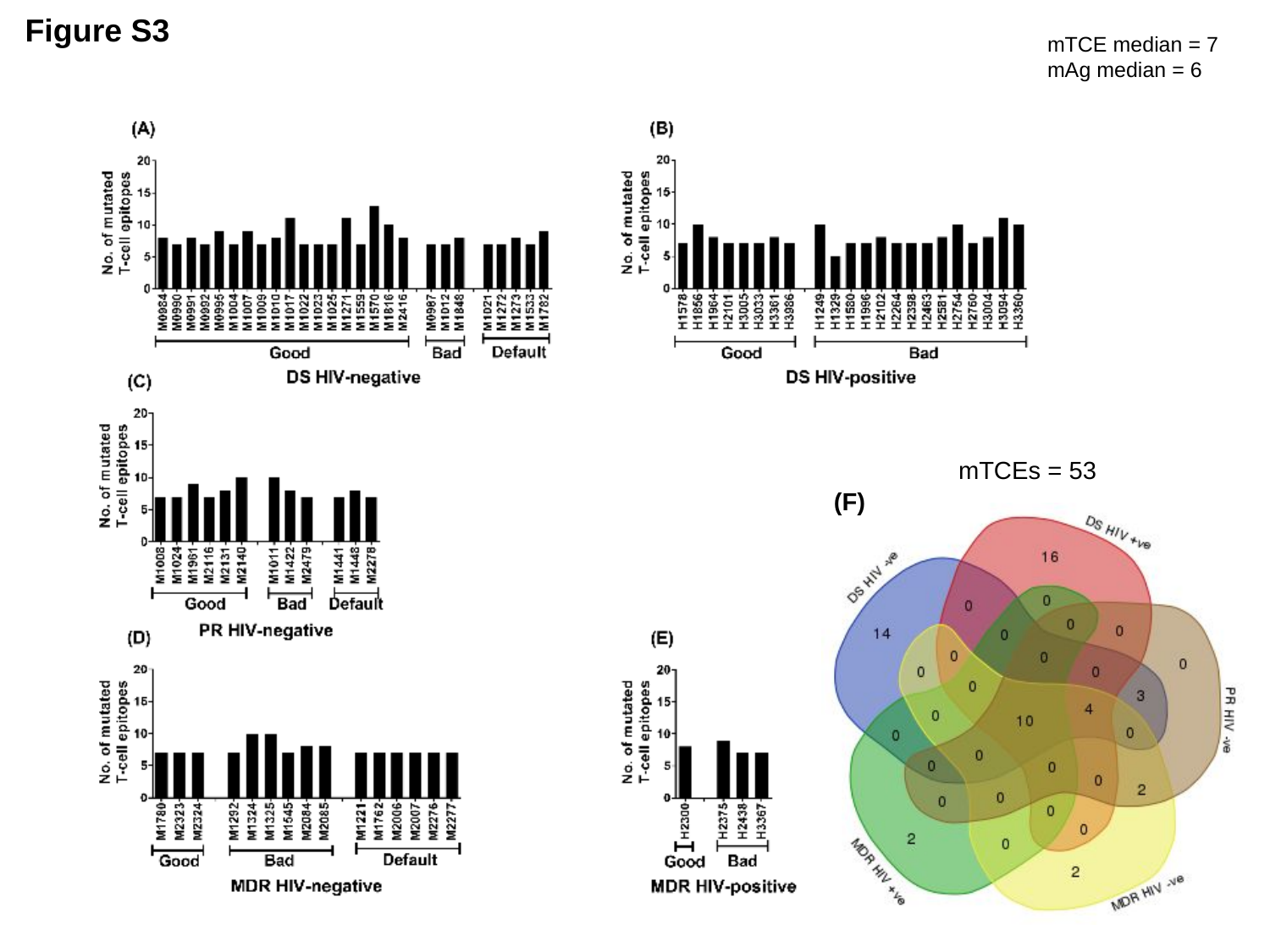

Figure S3
mTCE median = 7
mAg median = 6
mTCEs = 53
(F)

## Slide 4
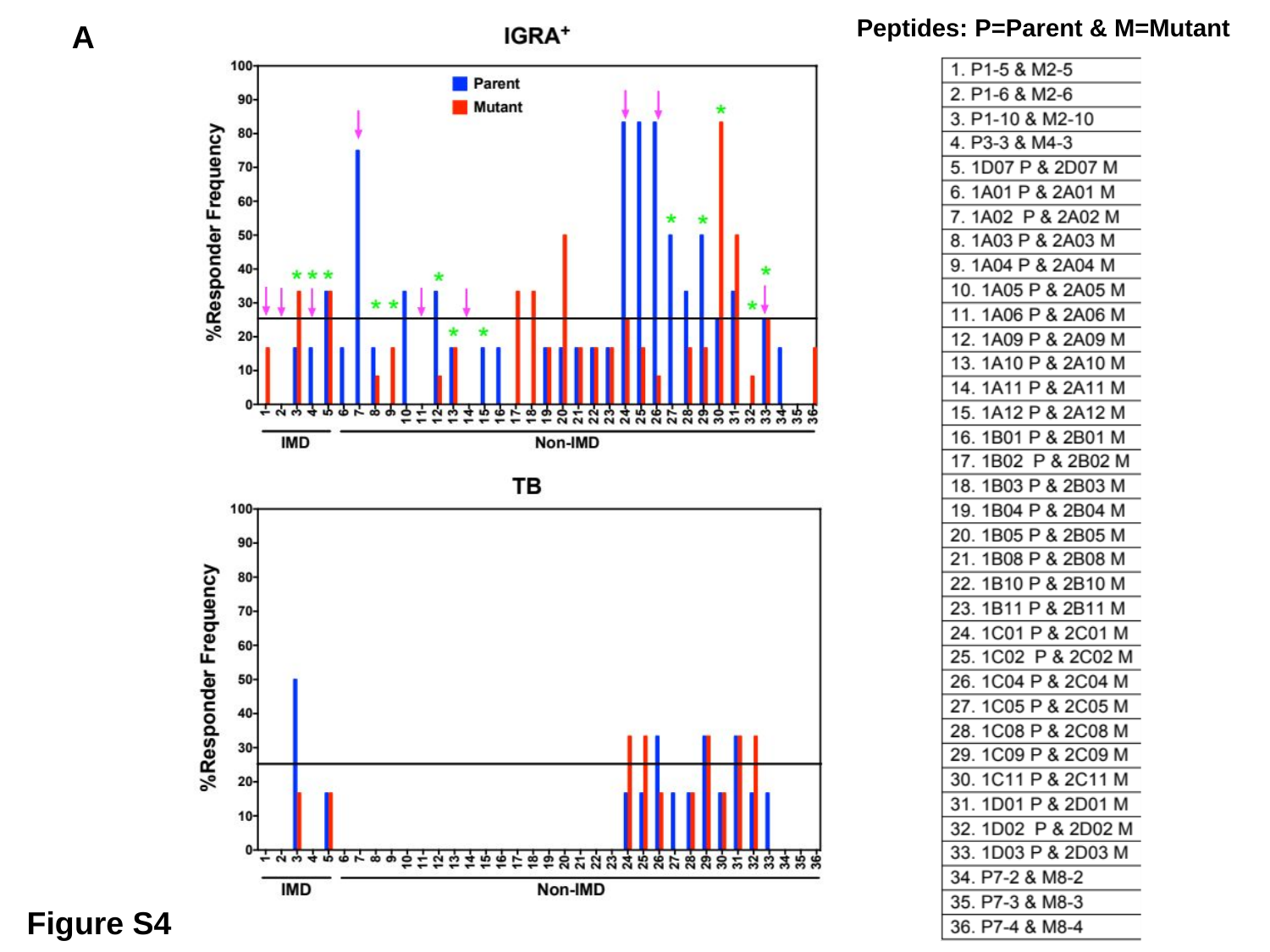

Peptides: P=Parent & M=Mutant
A
Figure S4

## Slide 5
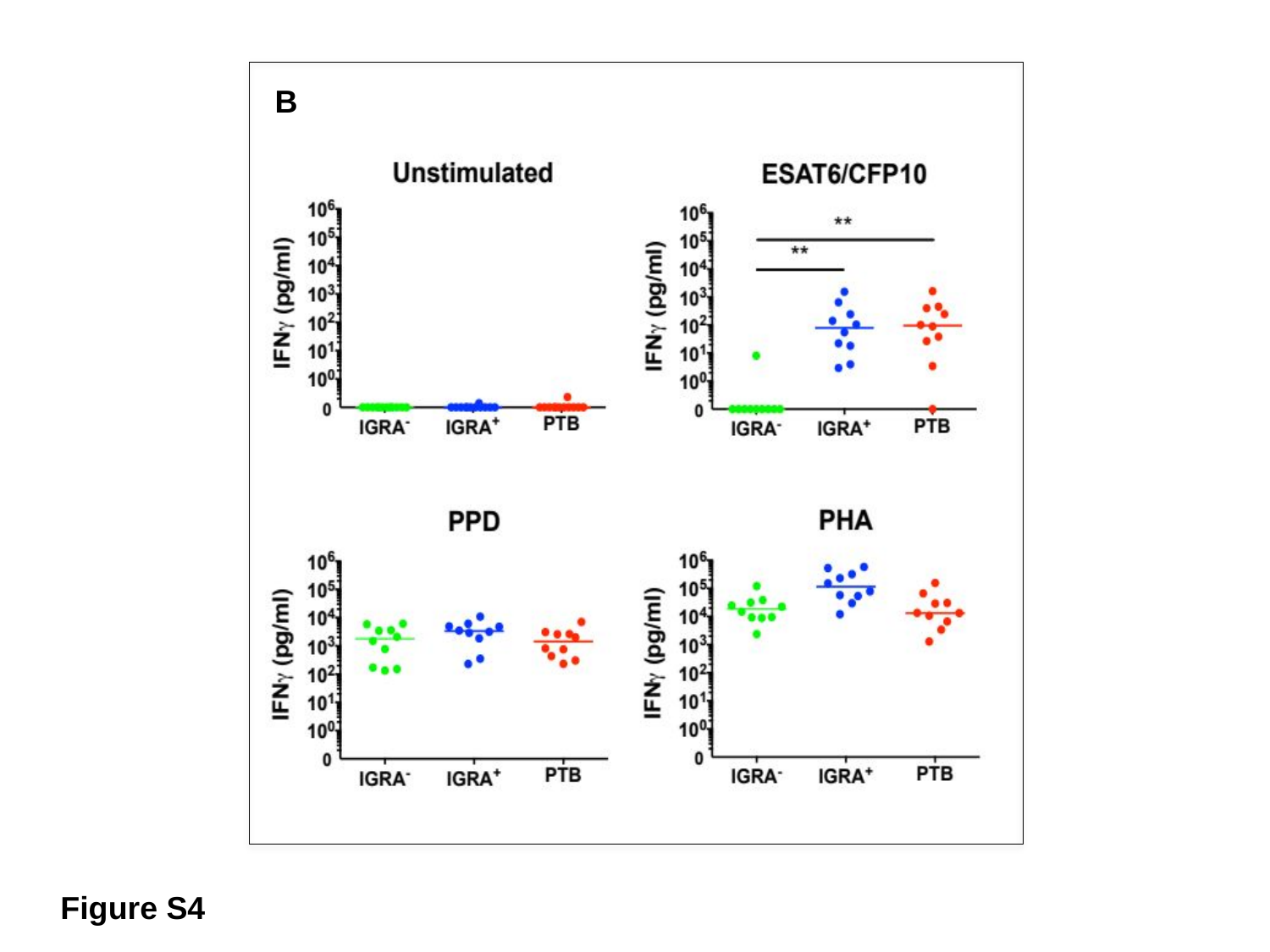

c
B
Figure S4

## Slide 6
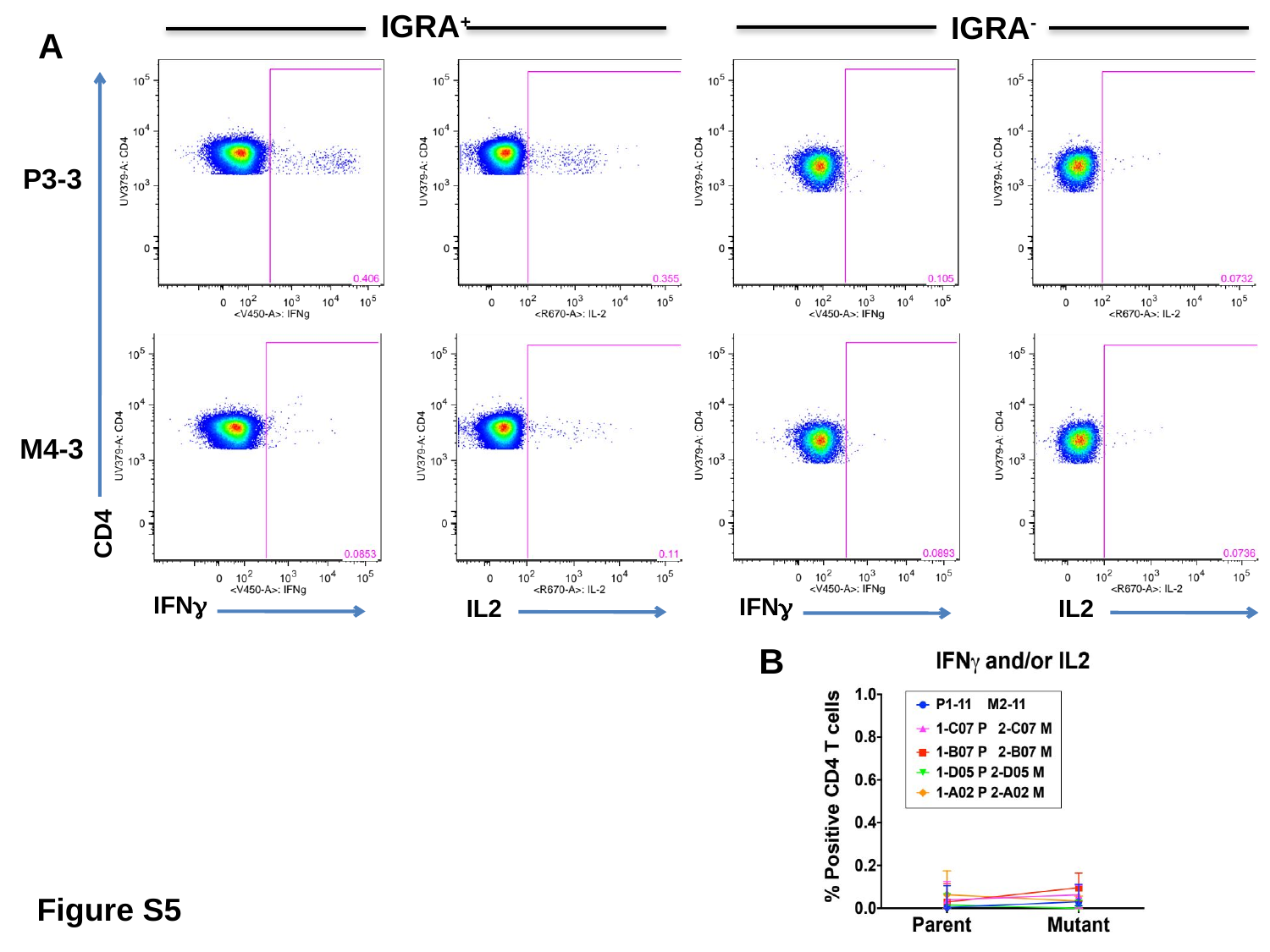

IGRA+
IGRA-
A
P3-3
M4-3
CD4
IFNg
IFNg
IL2
IL2
B
Figure S5

## Slide 7
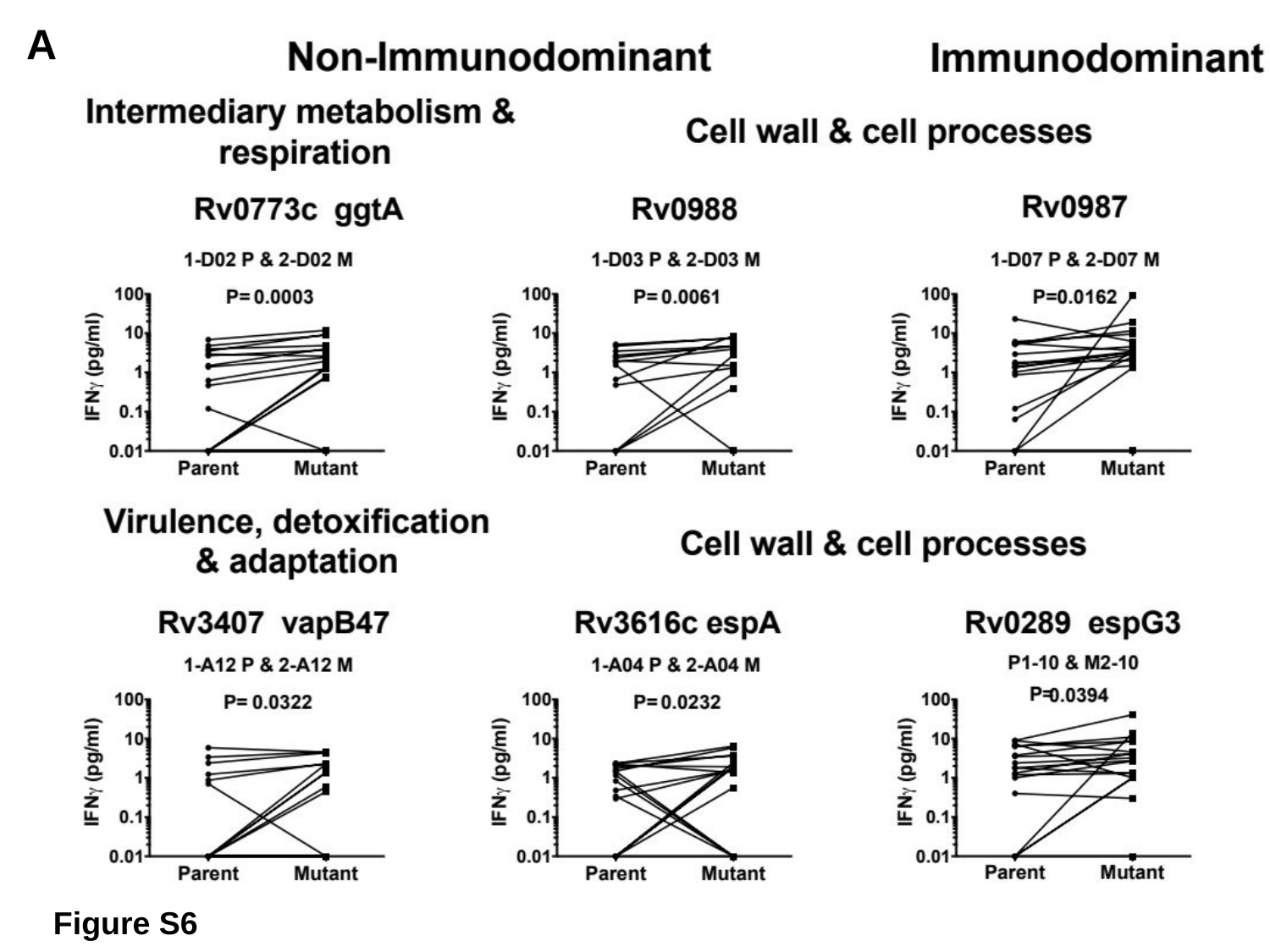

A
Figure S6

## Slide 8
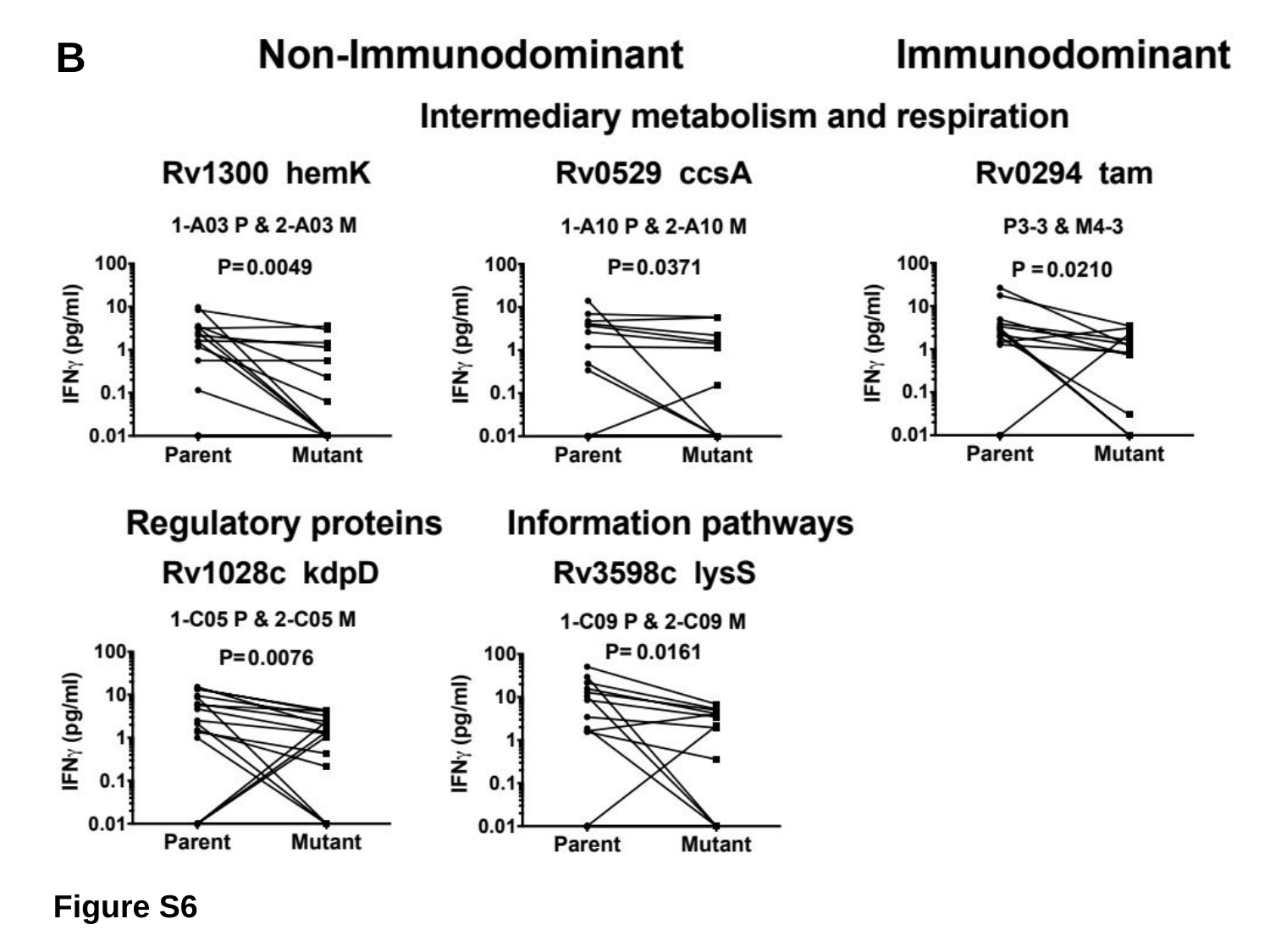

B
Figure S6

## Slide 9
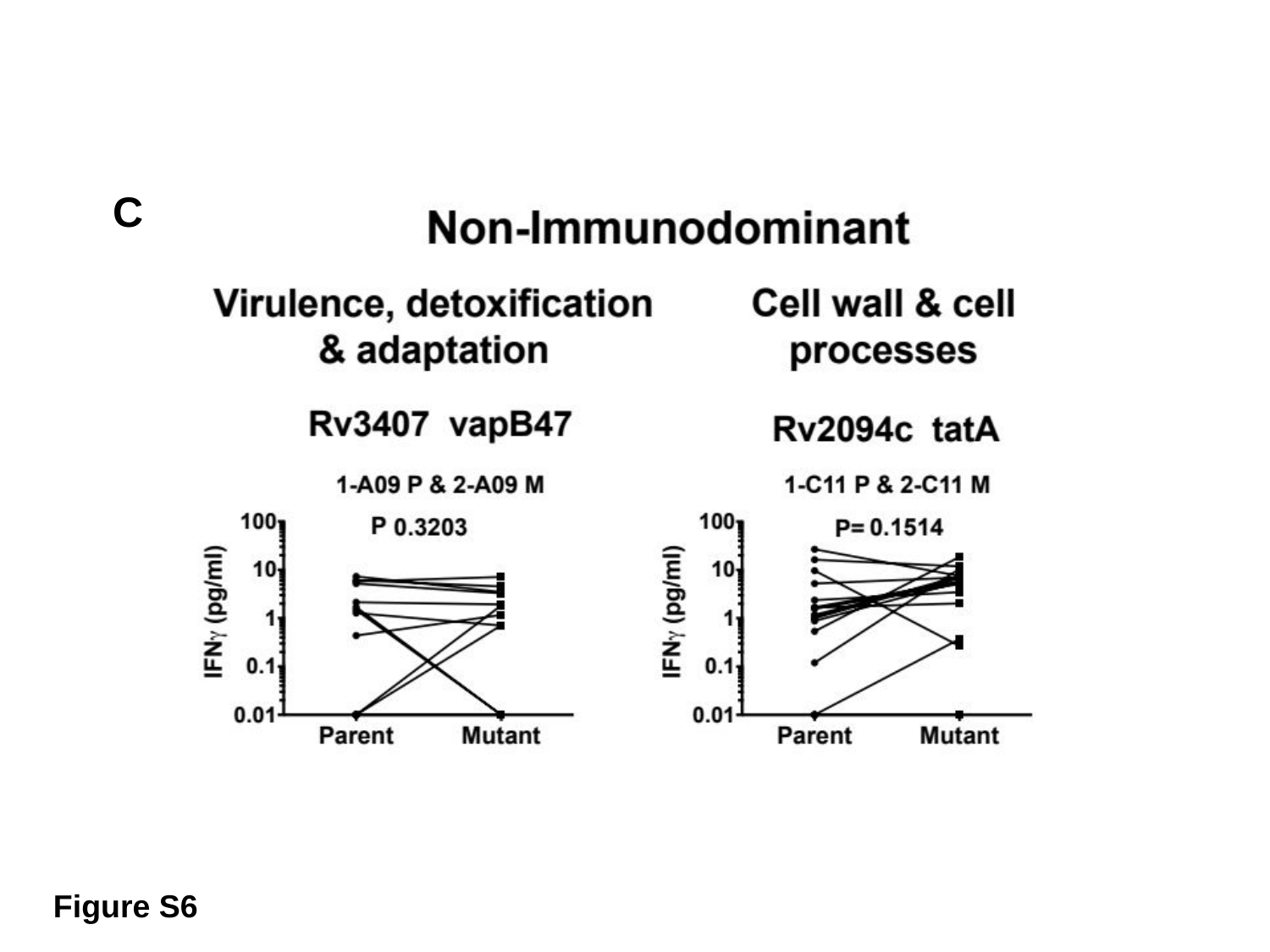

C
Figure S6

## Slide 10
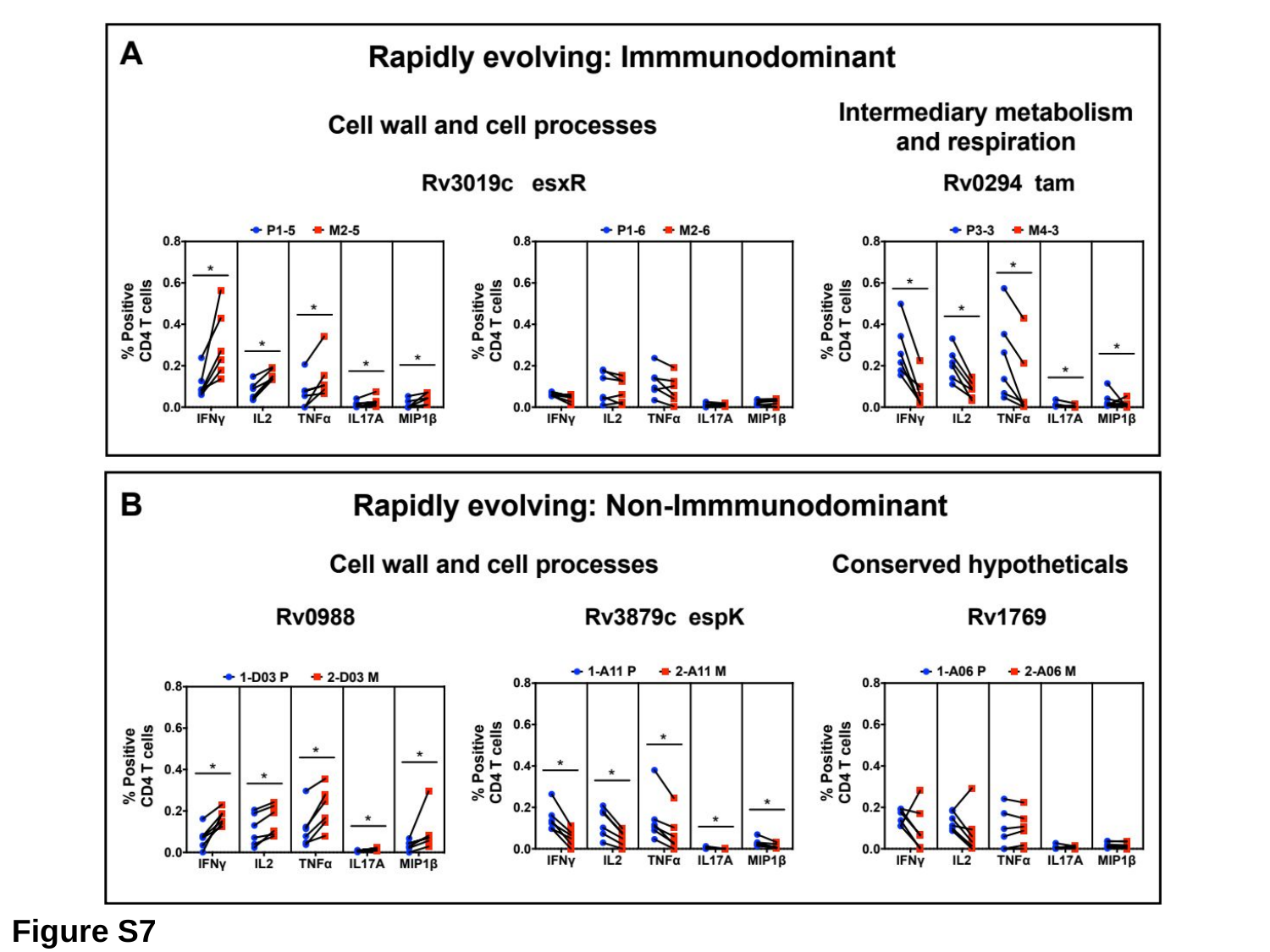

Figure S7

## Slide 11
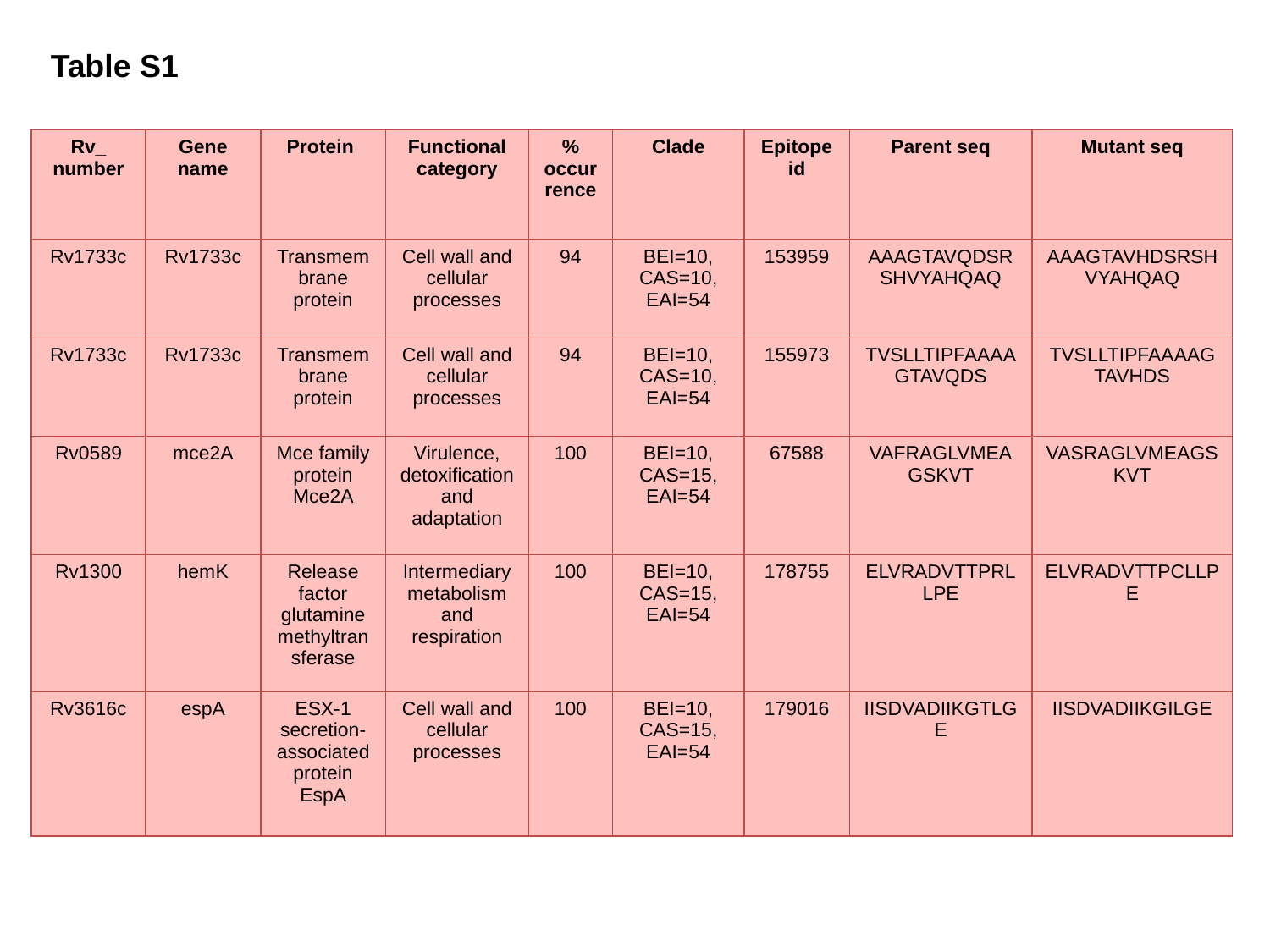

Table S1
| Rv\_ number | Gene name | Protein | Functional category | % occurrence | Clade | Epitope id | Parent seq | Mutant seq |
| --- | --- | --- | --- | --- | --- | --- | --- | --- |
| Rv1733c | Rv1733c | Transmembrane protein | Cell wall and cellular processes | 94 | BEI=10, CAS=10, EAI=54 | 153959 | AAAGTAVQDSRSHVYAHQAQ | AAAGTAVHDSRSHVYAHQAQ |
| Rv1733c | Rv1733c | Transmembrane protein | Cell wall and cellular processes | 94 | BEI=10, CAS=10, EAI=54 | 155973 | TVSLLTIPFAAAAGTAVQDS | TVSLLTIPFAAAAGTAVHDS |
| Rv0589 | mce2A | Mce family protein Mce2A | Virulence, detoxification and adaptation | 100 | BEI=10, CAS=15, EAI=54 | 67588 | VAFRAGLVMEAGSKVT | VASRAGLVMEAGSKVT |
| Rv1300 | hemK | Release factor glutamine methyltransferase | Intermediary metabolism and respiration | 100 | BEI=10, CAS=15, EAI=54 | 178755 | ELVRADVTTPRLLPE | ELVRADVTTPCLLPE |
| Rv3616c | espA | ESX-1 secretion-associated protein EspA | Cell wall and cellular processes | 100 | BEI=10, CAS=15, EAI=54 | 179016 | IISDVADIIKGTLGE | IISDVADIIKGILGE |

## Slide 12
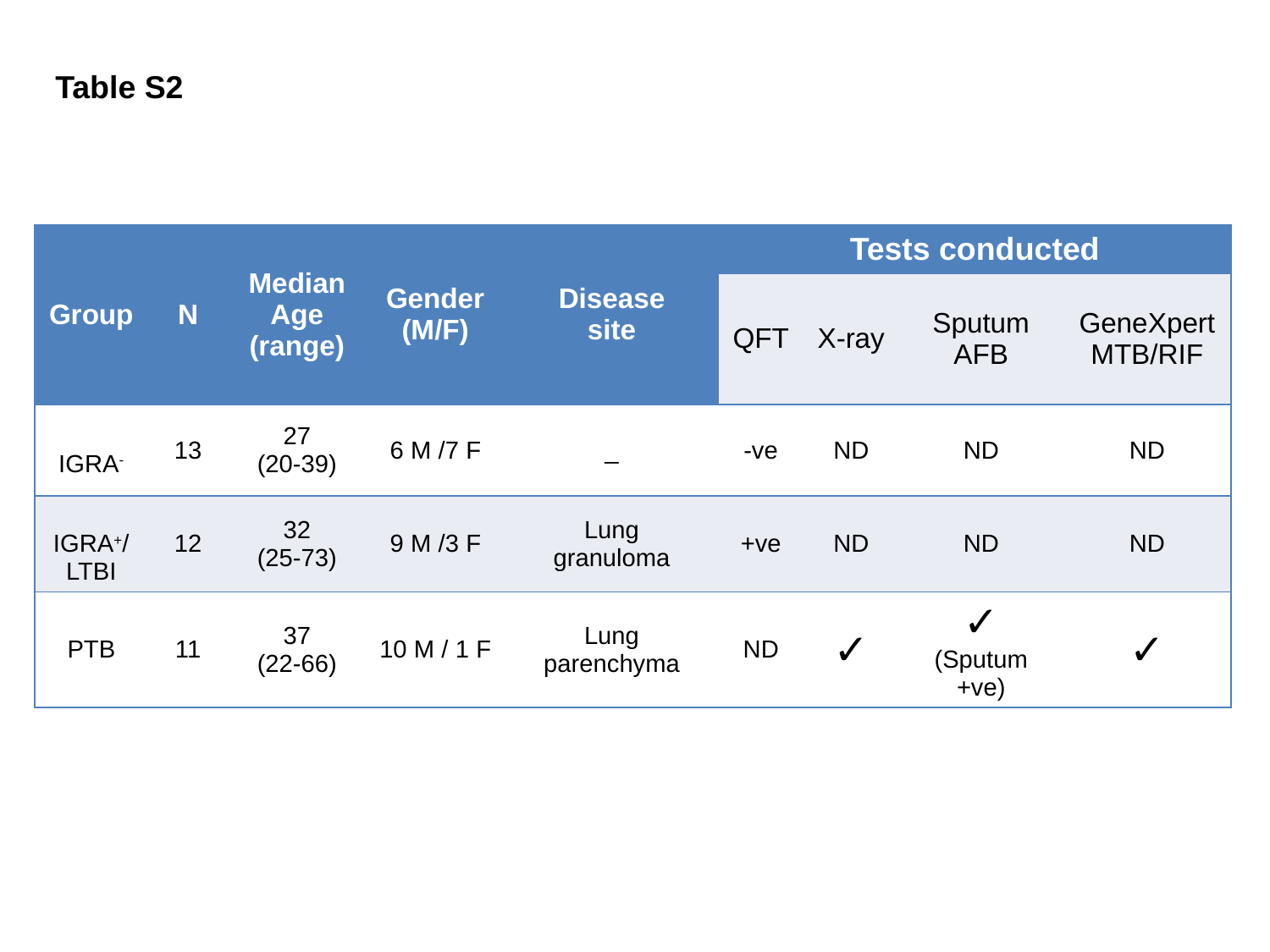

Table S2
| Group | N | Median Age (range) | Gender (M/F) | Disease site | Tests conducted | | | |
| --- | --- | --- | --- | --- | --- | --- | --- | --- |
| | | | | | QFT | X-ray | Sputum AFB | GeneXpert MTB/RIF |
| IGRA- | 13 | 27 (20-39) | 6 M /7 F | \_ | -ve | ND | ND | ND |
| IGRA+/ LTBI | 12 | 32 (25-73) | 9 M /3 F | Lung granuloma | +ve | ND | ND | ND |
| PTB | 11 | 37 (22-66) | 10 M / 1 F | Lung parenchyma | ND | ✓ | ✓ (Sputum +ve) | ✓ |
